# Long-Range Electrostatics in Serine Proteases: Machine Learning-Driven Reaction Sampling Yields Insights for Enzyme Design

**DOI:** 10.1021/acs.jcim.4c01827

**Published:** 2025-02-10

**Authors:** Alexander Zlobin, Valentina Maslova, Julia Beliaeva, Jens Meiler, Andrey Golovin

**Affiliations:** † Institute for Drug Discovery, Leipzig University Medical School, Brüderstraße 34, Leipzig 04103, Germany; ‡ Faculty of Bioengineering and Bioinformatics, Lomonosov Moscow State University, Leninskie Gory 1, building 73, Moscow 119234, Russia; § Institute for Medical Physics and Biophysics, Leipzig University Medical School, Härtelstr. 16-18, Leipzig 04107, Germany; ∥ Department of Chemistry, Vanderbilt University, 1234 Stevenson Center Lane, Nashville, Tennessee 37240, United States; ⊥ Center for Structural Biology, Vanderbilt University, PMB 407917, Nashville, Tennessee 37240-7917, United States; # Center for Scalable Data Analytics and Artificial Intelligence (ScaDS.AI), Leipzig 04081, Germany; ∇ Shemyakin and Ovchinnikov Institute of Bioorganic Chemistry, Russian Academy of Sciences, Miklukho-Maklaya 16/10, Moscow 117997, Russia; ○ Belozersky Institute of Physico-Chemical Biology, Lomonosov Moscow State University, Leninskie Gory 1, building 40, Moscow 119992, Russia

## Abstract

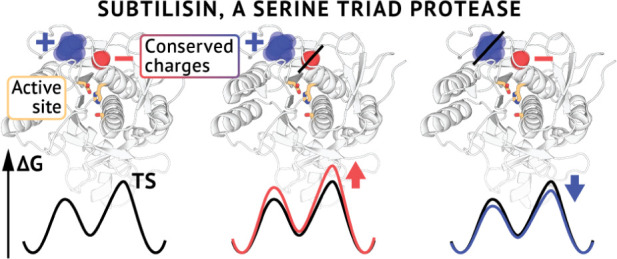

Computational enzyme design is a promising technique for producing novel enzymes for industrial and clinical needs. A key challenge that this technique faces is to consistently achieve the desired activity. Fundamental studies of natural enzymes revealed critical contributions from second-shell – and even more distant – residues to their remarkable efficiency. In particular, such residues organize the internal electrostatic field to promote the reaction. Engineering such fields computationally proved to be a promising strategy, which, however, has some limitations. Charged residues necessarily form specific patterns of local interactions that may be exploited for structural integrity. As a result, it is impossible to probe the electrostatic field alone by substituting amino acids. We hypothesize that an approach that isolates the influences of residues’ charges from other influences could yield deeper insights. We use molecular modeling with AI-enhanced QM/MM reaction sampling to implement such an approach and apply it to a model serine protease subtilisin. We find that the negative charge 8 Å away from the catalytic site is crucial to achieving the enzyme’s catalytic efficiency, contributing more than 2 kcal/mol to lowering the barrier. In contrast, a positive charge from the second-closest charged residue opposes the efficiency of the reaction by raising the barrier by 0.8 kcal/mol. This result invites discussion into the role of this residue and trade-offs that might have taken place in the evolution of such enzymes. Our approach is transferable and can help investigate the evolution of electrostatic preorganization in other enzymes. We believe that the study and engineering of electrostatic fields in enzymes is a promising direction to advance both fundamental and applied enzymology and lead to the design of new powerful biocatalysts.

## Introduction

Enzymes are efficient biological catalysts that are central to evolved life. They speed up chemical reactions by orders of magnitude in mild conditions, are highly specific and selective, and provide ways to regulate their activity in real time. These features make them desirable for applications in industry and medicine. Consequently, robust and affordable strategies to develop better or new enzymes are in high demand. Computational enzyme design is one of them. Despite much progress in the field, achieved turnovers in *de novo* design remain limited,^1−3^ as well as the predictive power in fine-tuning tasks. In the former case, the state-of-the-art approaches still yield products with activities orders of magnitude lower than natural or desired. As a consequence, computationally designed enzymes typically need to be optimized by directed evolution or other engineering techniques that largely alter second-shell residues.

It was suggested that the limitations of current computational strategies may arise from an insufficient consideration of the factors contributing to natural enzymes’ catalytic efficiencies.^4^*De novo* design approaches in particular treat enzymes usually in a reductionist way. The bulk of enzyme structure–function relationships is boiled down to the concept of a theozyme – a transition state (TS)-stabilizing arrangement of catalytic residues in space. A design objective is formulated as achieving this precise positioning. Shortcomings of produced enzymes are sometimes explained by insufficient accuracy in doing that.^5,6^ However, on multiple occasions, it was demonstrated that the outer shells of amino acids – circling the active site but not in direct contact with it – also play a major role in optimizing its efficiency. Conserved residues in these shells were shown to play major roles in influencing the correct positioning of the substrate^7,8^ and the hydration of the binding site,^9^ enhancing productive motions^10^ and ensuring the integrity of the catalytic site.^11^ The most attention, however, was drawn to the local electrostatic field influenced by these outer-shell residues.^12^ After the preorganization of the local interaction pattern around the TS,^13^ electrostatic field preorganization is now considered to be the second most important feature of biocatalysis. It was shown that polar residues as far as 1.5 nm away from the catalytic site contribute significantly to establish the field that would favor the reaction course.^14^ This notion gained traction in the suggested strategies for rational enzyme design.^15^ Some variants improved by directed evolution were found to do so by affecting outer shell electrostatics as well.^16^ Rational incorporation of such ideas improved *de novo*-designed Kemp eliminases with minimal intervention.^17^ The evidence is amassed that a better understanding of the evolution of the electrostatic preorganization and the development of new tools hold promise for enzyme design. However, the informed engineering of electrostatic fields in enzymes has yet to become a widely adopted strategy.

To date, evolutionary roles of outer-shell residues in natural enzymes were mostly scrutinized via *in silico* or *in vitro* mutagenesis to other identities.^12^ The interactions these residues form are short- and long-range, meaning that amino acids are tightly coupled in possibly very complex clusters within their shell. Point mutations to probe the role of charge would also affect such couplings, obscuring the interpretation of the results. Charged residues in the proteinogenic alphabets carry along unique capacities – and even requirements – to form h-bonds that are uncharacteristic of any other residues. This feature, however, is not mandatory to the general chemical space of amino acids.^18^ With the advances of synthetic biology,^19^ it may be possible to expand amino acid alphabets of life, redefining what a proteinogenic amino acid is.^20^ This could allow, for instance, the introduction of residues that are charged but hydrophobic. To fully harness the promise of synthetic biology, being able to decouple local and nonlocal effects of charged residues on enzyme function is therefore necessary. Disentangling these effects could also facilitate easier translation of the insight from enzyme evolution into actionable design hypotheses.

Herein, we wanted to probe a computational approach to study the effects of the evolved electrostatic field in enzymes decoupled from the local interaction profiles of individual residues. We worked with the industrially relevant^21,22^ serine triad hydrolases, for which only limited information on the role of second-shell and remote charged residues had been previously gathered. For a model triad protease, subtilisin Carlsberg, we identified conserved charged residues near the active site that do not directly interact with catalytic residues. We focused our attention on the closest two, Asp60 and Lys94. We used molecular modeling and QM/MM MD reaction sampling to uncover that the charges contributed by these two residues have opposite effects on the efficiency of the chemical step. The negative charge from Asp60 was found to greatly promote the reaction, while the positive charge from Lys94 slightly hindered it. Taking Asp60 into account was therefore found to be indispensable to understand subtilisin’s structure–function-evolution relationships. Moreover, our results show the usefulness of engineering a beneficial charge background to achieve native-like activities in *de novo* designed enzymes.

## Results

### Asp60 and Lys94 Are Evolutionary Conserved Charged Outer-Shell Residues of Subtilisin Carlsberg

The electrostatic field exerted by the whole structure of an enzyme plays an important role in its function as part of the phenomenon of catalytic preorganization. We speculated that tangible outcomes of this could be found as evolved stable localizations of charged residues near the catalytic site, but not as a part of it.

**Figure 1:**
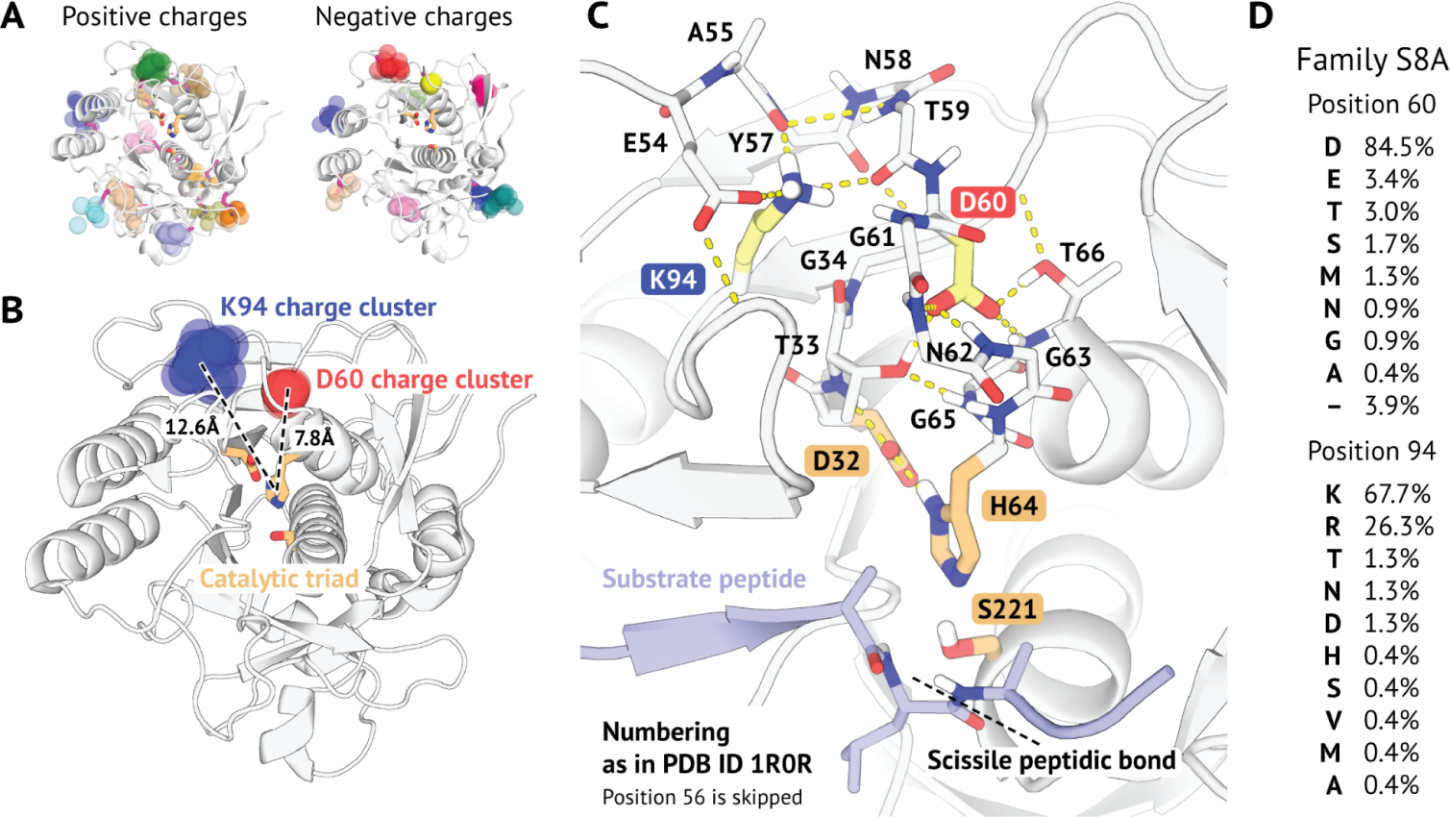
Conserved charged residues in subtilisin-like enzymes and their identity and interactions in subtilisin Carlsberg. (A) Bioinformatic analysis yields conserved locations of charged groups in structurally similar enzymes. (B) Conserved charge centers in subtilisin Carlsberg closest to the catalytic triad correspond to residues Asp60 and Lys94. (C) An overview of the enzyme’s active site. Asp60 and Lys94 (yellow) participate in extensive h-bonding networks. The catalytic triad is colored orange, and a substrate peptide is shown in blue. Nonpolar hydrogen atoms are omitted for clarity. (D) Conservation of positions 60 and 94 in the S8A family. Position numbering corresponding to PDB entry 1R0R is employed herein and throughout the paper.

To identify cases of evolutionary conserved charge clusters, we performed bioinformatic analysis of the structure of the serine triad protease subtilisin Carlsberg (Figure 1A). A nonredundant set of related structures was superimposed to identify N positive and D negative locations of conserved noncatalytic charged residues. Two locations closest to the catalytic triad were taken for subsequent analysis. They correspond to a conserved localization of a negative charge, encoded in subtilisin as Asp60 (PDB 1R0R numbering), and a positive charge coming from Lys94 (Figure 1B). Asp60 is located 7.8 Å away from the triad, while Lys94 is positioned farther at a distance of 12.6 Å. To get insights into the potential structural role of these residues, we analyzed their involvement in noncovalent interactions. Both residues formed extensive h-bonding networks reaching toward the catalytic triad (Figure 1C). To get additional evolutionary information, we analyzed a sequence alignment of the whole corresponding S8A family. We found that the identities that positions 60 and 94 assume in subtilisin correspond to the family evolutionary consensus (Figure 1D). Aspartate was found at position 60 in 84.5% of the sequences. Lysine at position 94 populated 67.7% followed by an also positively charged arginine with a population of 26.3%. Together these findings suggest that these charged residues were evolutionarily selected to play important roles in the enzyme’s function.

### Charges Close to the Active Site Have Contrasting Influence on the Reaction Kinetics

We sought to illuminate the reasons for the high evolutionary conservation of charges at positions 60 and 94. We were primarily interested in how they influence the chemical stage of the enzyme cycle since its efficiency is a primary objective of the evolutionary process for most enzymes. While neither of these two residues is necessary for catalysis, they might be indispensable in achieving the evolutionary achieved turnover rates. Therefore, we investigated how the reaction course would change quantitatively if there was no corresponding charge at positions 60 or 94, but the structure would otherwise be intact. This is difficult to achieve by amino acid substitutions because that would also change the local interaction pattern, thus influencing the measurables. That is why here we suggested a way to use molecular modeling to achieve the stated goal.

We used QM/MM simulations to model the reaction. Asp60 and Lys94 were kept in the MM region. We rescaled their partial charges to remove the net charge but preserve polarity and applied restraints to ensure reference positioning and interaction profiles. We used QM/MM MD with OPES enhanced sampling to model the reaction as a process, dynamically visiting each stable state and crossing each barrier multiple times. This was indispensable to collect statistics necessary to be sure of the significance of the potentially small observed differences. The other requirement for quantitatively interpretable free energy profiles was the nondegeneracy of the collective variable (CV) in the regions to which transition states were mapped. With a degenerate CV, alongside the transition state ensemble, some other distinct but more stable configurations are mapped to the same region of the variable. It effectively means that the CV cannot distinguish TS from other configurations that the system may realistically adopt.^23^ Naturally, a free energy profile along such a variable would be a poor representation of the true kinetics of the reaction. To approach building a nondegenerate CV, we trained DeepTDA data-driven collective variables for both reaction stages.

**Figure 2:**
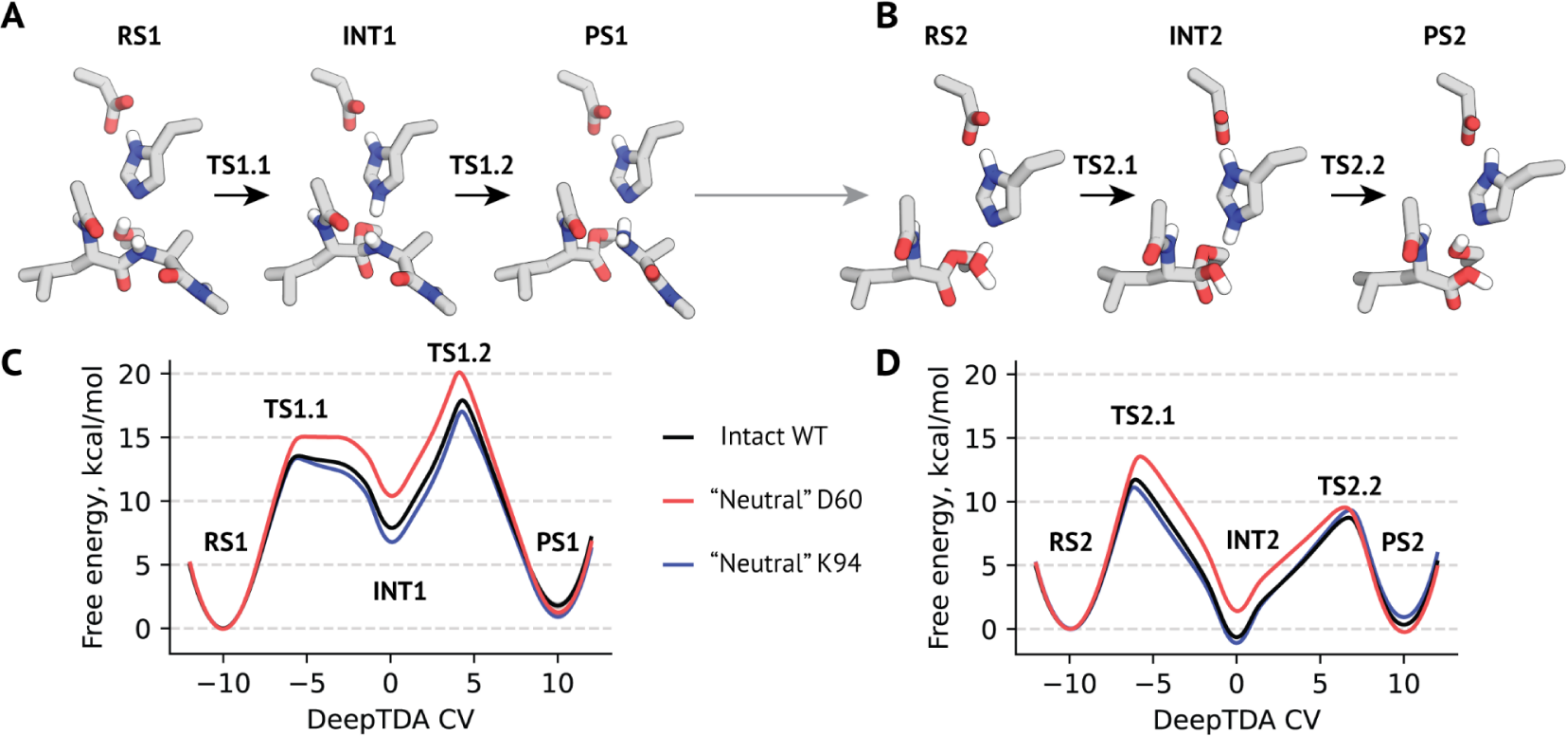
DeepTDA-powered reaction sampling quantifies the influence of conserved remote charged residues closest to the active site of subtilisin Carlsberg. (A) Reaction mechanism of the first (acylation) stage of the reaction catalyzed by subtilisin. (B) Reaction mechanism of the second (deacylation) stage. (C) Free energy profiles of the acylation stage in the intact model and with partial charges for Asp60 and Lys94 side chains shifted to yield net zero charge (Table S1). (D) Free energy profiles of the deacylation stage for the same models as in (C). Standard errors of means are shown but are often smaller than line widths. For their values refer to Figure S1.

We applied machine-learning-enhanced sampling to successfully model both reaction stages of subtilisin Carlsberg (Figure 2). The limiting stage was found to be the second step of the acylation process, in line with previous results.^24−26^ The estimated activation barrier for the intact enzyme model was 17.9 kcal/mol. With the net charge on Asp60 nullified, energy levels of both transition states and intermediates of the acylation stage were significantly raised (Figure 2C and Table 1). The same was found to be true for the first transition state and the intermediate of the deacylation stage (Figure 2D). The estimate of the barrier at the limiting stage was elevated by ∼2.3 kcal/mol with respect to the unperturbed enzyme. “Neutral” Lys94 model behaved much more alike the reference, with a notable exception for the limiting stage. Deleting the charge on Lys94 resulted in ∼0.8 kcal/mol decrease in the activation free energy compared to the intact enzyme.

**Table 1:** Calculated Free Energy Parameters of the Reaction Catalyzed by Subtilisin Carlsberg^a^

	Acylation stage Δ*G* (kcal/mol)			Deacylation stage Δ*G* (kcal/mol)		
	Intact	“Neutral” D60	“Neutral” K94	Intact	“Neutral” D60	“Neutral” K94
RS → TS1	13.5 ± 0.1	15.12 ± 0.06	13.4 ± 0.1	11.7 ± 0.2	13.6 ± 0.1	11.1 ± 0.1
RS → INT	7.9 ± 0.1	10.4 ± 0.1	6.8 ± 0.1	–0.64 ± 0.06	1.4 ± 0.1	–1.1 ± 0.3
RS → TS2	17.9 ± 0.1	20.2 ± 0.1	17.1 ± 0.1	8.74 ± 0.04	9.56 ± 0.09	9.3 ± 0.2
RS → PS	1.8 ± 0.3	1.3 ± 0.4	0.9 ± 0.2	0.33 ± 0.09	–0.2 ± 0.2	0.9 ± 0.3

a “Neutral” forms correspond to systems in which partial charges for the side chain of the respective residue are shifted to yield net zero total charge (Tables S1 and S2).

**Figure 3:**
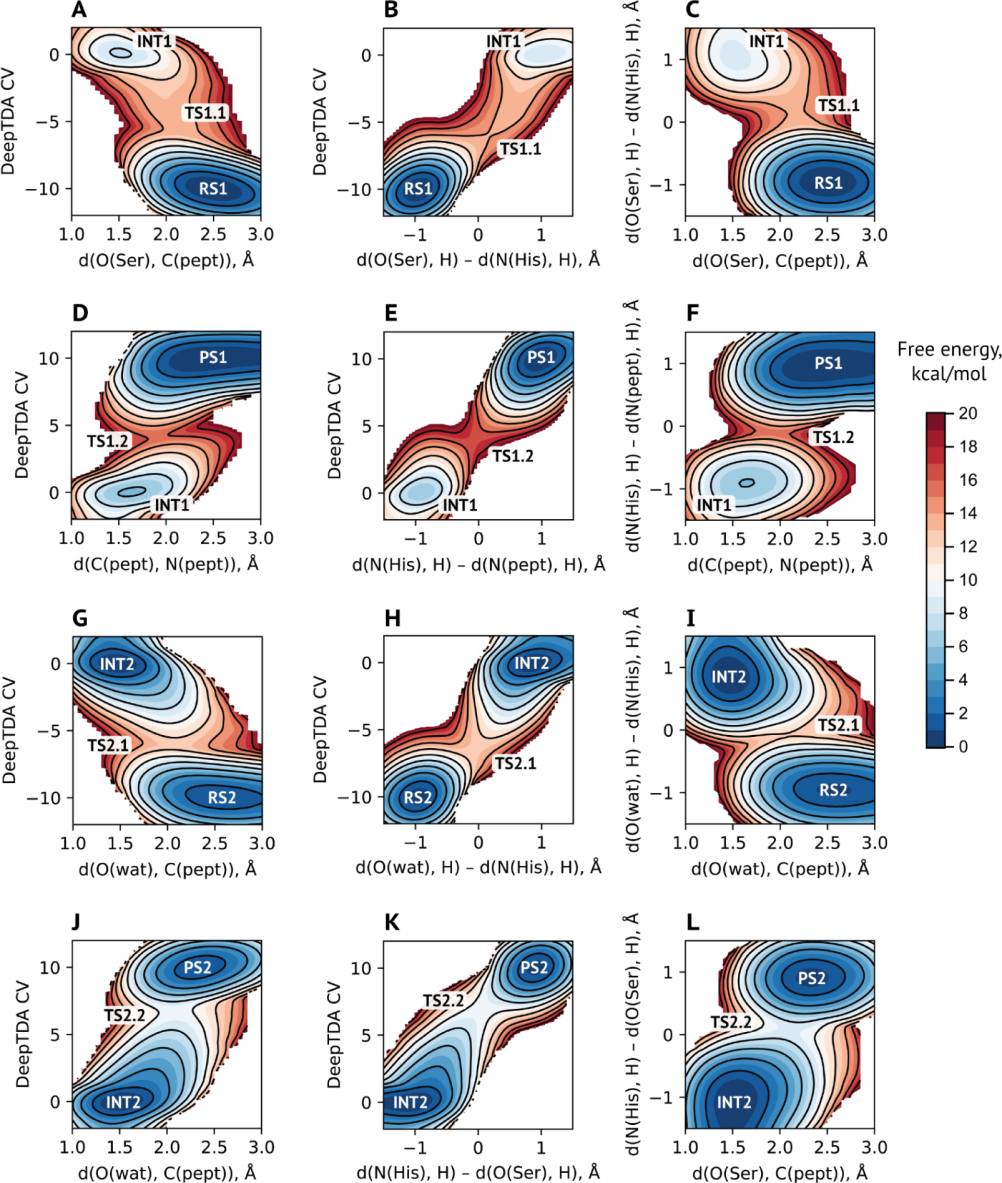
Physical meaning and TS nondegeneracy of the learned DeepTDA variables. Shown are reweighted profiles for the intact enzyme. (A–C) First step of the acylation reaction stage. (D,E) Second step of the acylation reaction stage. (G–I) First step of the deacylation reaction stage. (J–L) Second step of the deacylation reaction stage. Ser = catalytic Ser221, His = catalytic His64, wat = attacking water molecule, and pept = substrate peptide. C (pept) = carbonyl oxygen of the substrate peptide’s Leu (P1′) residue, N(pept) = peptide group nitrogen of the substrate peptide’s Ala (P1) residue. State labels correspond to those from Figure 2A,B.

To ensure the reliability of the obtained energy estimates, we assessed whether the DeepTDA CV maps transition states to the unique regions in the CV space. For that, we reweighted the values of the CV against rational variables associated with the chemical transformations (Figure 3). We found that for all reaction stages our CVs indeed mapped specific regions to transition state configurations in a nondegenerate way. TS-associated maxima from 1D profiles were found to represent chemically viable transition configurations expressed as rational variables. No configurations with free energy lower than that of TS were mapped to the TS-associated regions of DeepTDA space. This supports the interpretability of the profiles and free energy differences obtained for our systems.

### Charge Alterations at Positions 60 and 94 Influence the Structure of the Enzyme and the Enzyme–Substrate Complex

Enzyme residue identities might be selected by the evolutionary process for multiple reasons, and optimizing the turnover is only one of such possibilities. Residues might be conserved because they ensure structural integrity in critical regions. For that, they form unique patterns of noncovalent interactions that might as well be unattainable by any of the other 19 amino acids. To investigate this possibility for positions 60 and 94, we performed classic MD simulations for both peptide-bound and peptide-free models of our systems. After 500 ns, we did not observe substantial general changes in backbone flexibility, unfolding events or peptide unbinding (Figures S2–S4). This was to be expected since the intervention was very limited. We, however, noticed pronounced local effects, such as stable changes in the catalytic His–Asp interaction geometry as well as in the conformation of loop 50–64 (Figures S5 and S6). We investigated these features in greater detail.

**Figure 4:**
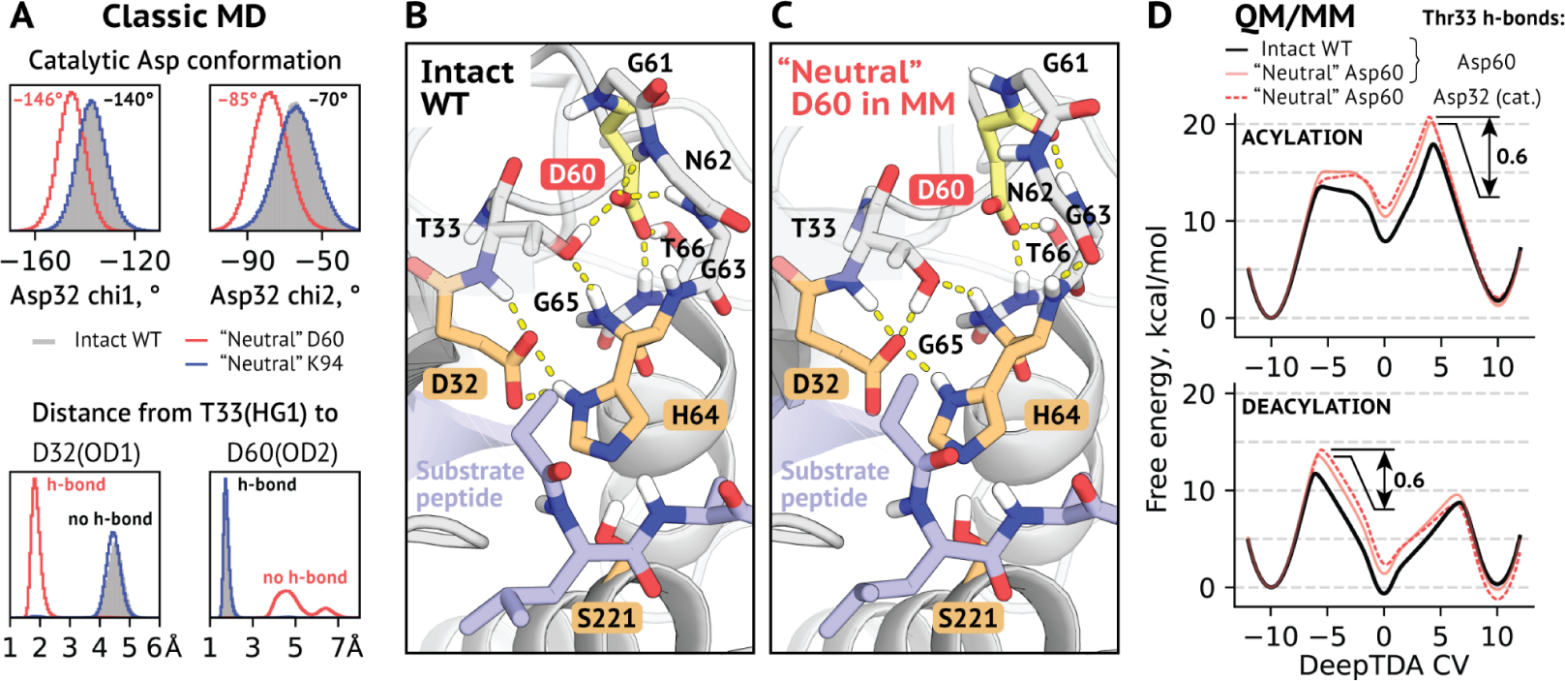
Disruption in the h-bonding network centered at the residue 60 affects subtilisin function through changes in the geometry of the catalytic Asp32. (A) Catalytic Asp32 geometry and h-bonding changes when the net charge on Asp60, but not on Lys94, is zero. (Upper graph) Distributions of the side chain rotation angles chi1 (N-CA-CB-CG) and chi2 (CA-CB-CG-OD1). (Lower graph) Distributions of the distance from Thr33 polar side chain hydrogen (HG1) to two potential h-bond acceptors (Asp32 OD1 and Asp60 OD2). Shown are distributions from the last 100 ns of each replica of enzyme–substrate complex simulations. (B) Original interaction geometry observed in the intact enzyme–substrate complex. Thr33 h-bonds Asp60 (yellow). (C) Altered interaction geometry was observed with the modified net charge for Asp60. Thr33 h-bonds catalytic Asp32. As in (B), nonpolar hydrogen atoms are omitted for clarity, the catalytic triad is shown in orange, the substrate peptide in blue. (D) Influence of the Thr33-Asp32 h-bond on the reaction efficiency. Profiles for systems with the presence of this h-bond are shown as dashed. (Upper graph) Free energy profiles for the acylation stage. (Lower graph) Free energy profiles for the deacylation stage. Standard errors of means are shown, but are often smaller than line widths.

Noticeable changes in the conformation of the catalytic Asp32 were observed in the system with “neutralized” Asp60 (Figure 4A). They were associated with Asp60 losing a number of h-bonds due to the lower charge on its side chain δ-oxygens (Figure 4B,C). Most importantly, Thr33 was found to switch to h-bond Asp32 instead, causing the observed changes in the geometry. This behavior was not modeled in reaction sampling experiments above since only the effect of charge was investigated but can potentially have an effect on the triad efficiency. To probe this, we lifted some restraints and allowed the replication of this behavior in QM/MM to find an additional increase in the activation free energy of 0.6 kcal/mol (Figure 4D and Table S5). Therefore, in the absence of conserved Asp60, having Thr at position 33 is detrimental, hinting on potentially high coevolution of the region. It is likely that Asp60 is crucial to the enzyme’s efficiency not only due to its charge but also to the unique propensity of this residue to form numerous strengthened charge-assisted h-bonds at once.

**Figure 5:**
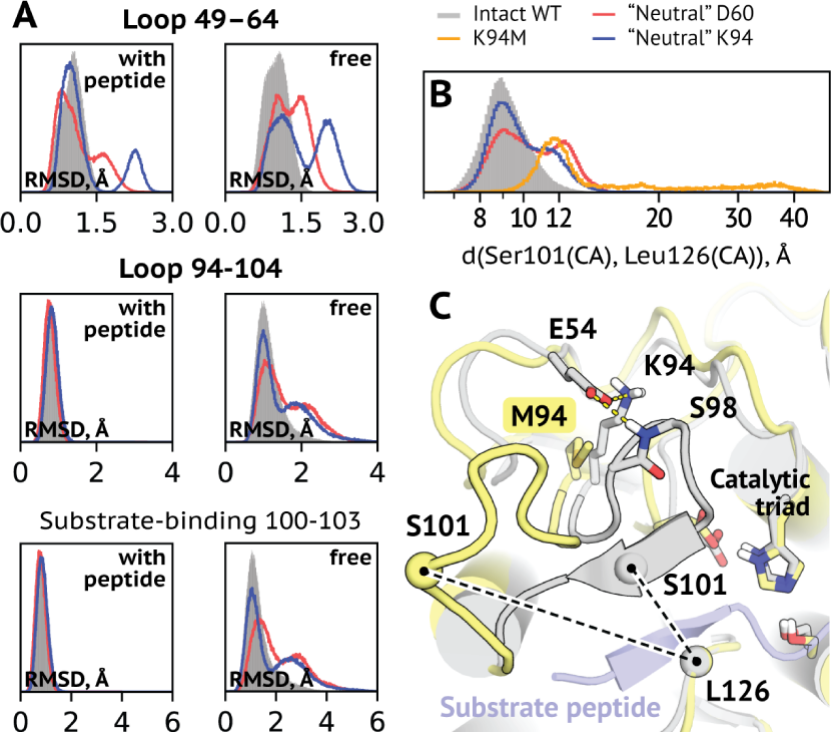
Disruptions in h-bonding networks around residues 60 and 94 affect the substrate-binding loop. (A) Deviations of backbone positions of loop regions that harbor Asp60 and Lys94 and interact with them. Substrate-binding region 100–103 (lower graph) is of particular interest since it forms a beta-sheet with the substrate (C) Aggregate RMSD values from the last 100 ns of each replica are shown. (B) Distribution of the width of the substrate-binding groove in free enzyme models. Shown are measures from the last 100 ns. The K94M system was modeled for 1.5 μs, others for 0.5 μs per replica. (C) Example of changes in the substrate-binding gorge in the K94M variant (shown in yellow) as compared to the WT (gray). Substrate peptide is shown for illustrative purposes and colored blue. Dashed lines correspond to the distance plotted in panel B.

This notion was further supported when we considered changes in the loops harboring – and supported by – residues Asp60 and Lys94. Charge scaling resulted in these loops assuming alternative conformations (Figure 5A). Since the peptide, once bound, acted as an interaction intermediary to stabilize regions of the protein, it was not surprising that free forms demonstrated this more prominently. We noticed changes in positions 100–102 that play a crucial role in substrate binding by forming a short beta-sheet with it. Lys94 influences the conformation of this region via an intermediary Glu54 (Figure 1C). Glu54 is not conserved within the S8A family (Table S6), with other, mostly polar residues occupying this position, which might mean that its charge alone is not significant for the reaction. We performed simulations for systems with the “neutral” Glu54 to confirm this. This residue is not positioned well to shield the active site from the influence of Lys94, which explains the almost absent effect of removing its charge.

We suggested that Lys94 is primarily conserved because of its propensity to donate multiple h-bonds that influence the stability of the substrate-binding gorge (Figure 5B), and not its charge. To probe this idea further, we modeled an h-bond-lacking K94M variant in a free form for 1.5 μs to find that the substrate-binding loop deviates even more from the evolutionary optimized geometry (Figure 5C). These results suggest that residues at both positions evolved to support local interaction patterns specific to their identities, and these interaction networks play a role, in particular, to facilitate efficient substrate binding.

## Discussion

**Figure 6:**
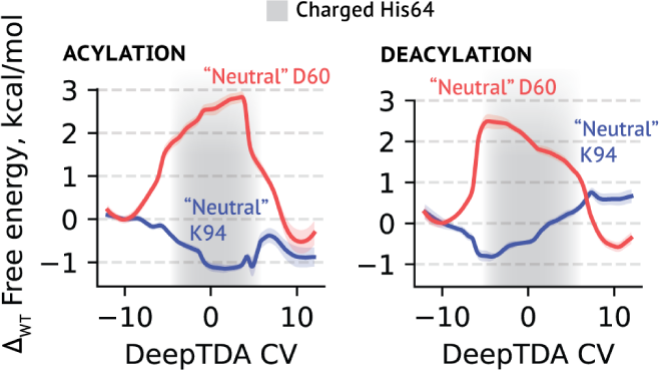
Changes in the outer-shell electrostatics might influence the catalytic efficiency through (de)stabilization of the catalytic His64. Shown is the difference between free energy profiles computed for the system with the “neutral” Asp60 (red) or Lys94 (blue) and the profile for the unperturbed system (Figure 2C,D). Shaded gray areas represent regions of the CV that correspond to the protonated cationic form of catalytic His64.

The importance of conserved noncatalytic residues in enzymes for their function is not always well established, which results in reductionist approaches to *de novo* enzyme design. In this work, we used molecular modeling to investigate the effect that conserved charged residues close to the active site might have on the catalytic efficiency of a model serine triad protease, subtilisin Carlsberg. We focused on computationally removing only the charge of the aforementioned residues while keeping the local interaction profiles intact. We implemented extensive deep-learning-assisted reaction sampling to quantitatively analyze the free energy profiles for both the acylation and deacylation stages of the reaction. Unprecedentedly low uncertainty in free energy evaluation allowed us to obtain confident differences in the activation free energies due to perturbations in the electrostatic field around the active site. We found that the negative charge from Asp60 located ∼8 Å away from the center of the catalytic site is crucial to achieve the enzyme’s catalytic efficiency with the associated contribution of 2.3 kcal/mol to lowering the barrier. The reason this negative charge is so important might be in the stabilization of the cationic His in both tetrahedral intermediate states (Figure 6). It results in slight changes in the most probable TS geometries, most pronounced for His-H distances (Table S7). In contrast, the positive charge from the second-closest charged residue, Lys94, opposes the efficiency of the reaction by raising the barrier by 0.8 kcal/mol, inviting the discussion into its role and trade-offs that might have taken place in the evolution of S8A family enzymes.

### Functional Roles and Evolution of Conserved Asp60 and Lys94

Our results suggest that the identity of position 94 is selected to ultimately stabilize the correct conformation of the substrate-binding gorge. It does so by acting as a local interaction hub, for which it is required to donate at least three h-bonds. There are only two residues that can achieve that, Lys and Arg. Therefore, it is not surprising that they are dominant at this position (Figure 1D). Their positive charge – found to be undesirable for the reaction process – may therefore be merely a confounding effect of the limitations of the natural amino acid space.^27^ Most prominent donors of hydrogen bonds in it are necessarily positively charged under neutral pH. We speculate that a long uncharged residue that could donate three or more hydrogen bonds may be better suited for this position. Such a residue can be introduced artificially. Advances in synthetic biology make such interventions ever more accessible, with works on expanded gene alphabets^28,29^ and aminoacyl synthetases.^30,31^ In this context, it is intriguing to consider what prospects the incorporation of noncanonical amino acids might have to improve already evolved enzymes beyond their natural activities. It is likely that many evolutionary trade-offs such as that presented here could be overcome to yield better catalysts for industry. This naturally calls to address the challenge of a systematic identification of such cases in other enzymes.

We showed that Asp60 plays a dual role. Its charge alone significantly enhances the processivity of the enzyme, while its immediate neighborhood’s structural integrity relies heavily on interactions with it. In its absence, Thr34 becomes mildly detrimental, implying coevolution. Interaction profiles (Figure 4B) with five h-bonds show that no other residue may be introduced at this position without requiring massive changes in its surroundings. It is unlikely that Asp was introduced to that structural context by chance. We speculate that selection for negative charge must have occurred first, with its structural context evolving afterward. In this context, it is intriguing to probe the evolutionary history of subtilisin-like proteases that may be approached by methods like ancestral sequence reconstruction.^32^

### Limitations and Outlook

With our results, we show promise in performing computational reverse-engineering to understand enzymes. We were successful in achieving our stated goal of decoupling the local and nonlocal effects of conserved outer-shell residues. Our approach may be transferred to other cases in a straightforward way. It was, however, limited by the description of the key residues with force fields relying on fixed point charges. It was shown that polarizable force fields achieve a better description of local electrostatic fields,^33^ and thus their application to this case might improve the results obtained herein.

Our approach relied on holistic sampling of the whole reaction process in multiple recrossings. It has a number of advantages, including a direct consideration of conformational heterogeneity and entropic contributions, statistical uncertainty estimation, and the ability to capture shifts in the TS geometry. Reaction sampling, after the CV and parameters are defined, proceeds automatically, limiting manual work, and the developed CV might be transferable to other S8A proteases. It comes at the price of limitations. Efficient reaction sampling is only possible once the reaction mechanism is well understood, which is a common starting point for *de novo* enzyme design but might not be so for a particular researcher. If so, a prior study is needed. Dynamical sampling is also costly, which limits the choice of the QM methods. The roles of Asp60 and Lys94 charges might have also been suggested by other methods, for example, ones focusing on estimating preferential stabilization of TS over RS through single-point QM/MM calculations.^34,35^

Our approach may also be used in reverse, by creating a charge instead of destroying it and probing its effect. If beneficial, then an enzyme design task is formulated to accommodate the charge-bearing residue. Within the presented framework, such a “charge screening” design operation can be implemented seamlessly. Its practical applicability remains to be addressed.

We scrutinized one particular family of serine triad hydrolases. It is yet unclear how transferable our findings are to, for example, chymotrypsin-like proteases^36^ or esterases. If they are not, then it would raise a question of evolutionary limitations that prevented the acquisition of beneficial charges in the vicinity of active sites. It is also intriguing to apply a bottom-up approach to analytically derive an electrostatic field beneficial for the reaction and compare it with evolutionary acquired features.^37^ As shown here, natural enzymes should not be expected to perfect this correspondence since multifactor optimization of their sequences would always require trade-offs. However, this comparison might allow for a systematic approach to easier reveal such trade-offs.

### The Promise of Data-Learned Collective Variables for Computational Enzymology

In this study, we pioneered the application of machine learning-powered collective variables to sample enzymatic reactions beyond just a proof-of-principle setup.^38^ Previously, such variables were successfully applied to other tasks including conformational transitions,^39^ crystal formation,^40,41^ sampling water rearrangement upon ligand binding,^42^ ligand dissociation,^43^ and the traversal of a small molecule through the membrane.^44^ Here, we correctly modeled multiminima enzymatic reaction paths with only 1D DeepTDA CVs. They demonstrated good discriminative ability, even without TS configurations in the training set. This allowed us to correctly estimate the energy at each TS without projection issues that more traditional variables have.^23,24^ Efficient sampling was also achieved, which led to low uncertainties and quick convergence. We note that the presented study of charge effects is only one example of the practical usefulness of machine learning-assisted QM/MM MD. Such approaches may be also used as an *in silico* assay within *de novo* enzyme design pipelines, screening hundreds of candidate variants on their way to experimental validation. A strength of this approach lies in its automatization. CVs and OPES parameters need to be devised once and afterward are reusable for any similar system with minimal manual work. Intriguing direction here is to develop universal collective variables able to sample, for instance, reactions in any serine triad hydrolases.

We note that well-discriminating variables are not always good at driving transitions, and *vice versa*. This is also not guaranteed for DeepTDA variables. While our models performed well in this regard, we speculate that even better performance might be achieved with path-like CVs that directly exploit information about the transition regions.^45,46^ Nevertheless, even the current approach and models may be potentially attractive as a computational assay to screen for catalytic activities. Sampling 100 ps per walker is likely redundant (Figure S7), and thus, it requires less than 12 CPU-hours per walker, opening a way to screen dozens and hundreds of enzyme variants on modern HPC setups. Advances in DFT-level accurate GP3-xTB, machine-learned corrections to DFTB,^47^ and foundational deep-learning potentials^48^ could increase the accuracy of the approach for enzymes with more challenging biochemistry.

### Importance of Considering Electrostatic Preorganization in Enzyme Design

Catalytic sites are usually understood as minimal sets of residues needed to observe any enzymatic activity. Here we showed that they alone cannot achieve native-like catalytic activities, with Asp60 crucial for subtilisin efficiency. Nevertheless, in computational *de novo* enzyme design studies, it is often considered sufficient to achieve a desired positioning of catalytic sites alone. Subsequent fine-tuning then may be focused mostly around creating a suitable substrate binding site. The state-of-the-art *de novo* serine hydrolase design study implied the same logic,^3^ was by design biomimetic (borrowing the natural chemistry), and its results still fell orders of magnitude from practically useful turnover numbers. We believe that practical needs may require introducing a novel (re)definition of the active site as a minimal functional motif needed to achieve a specified percentage of the native-like activity. Subsequent reassessment of enzymology and building of new databases might greatly advance our fundamental understanding of both enzymes and enzyme design.

## Methods

### Bioinformatic Analysis

We constructed our set of representative structures of subtilisin-like enzymes as follows. PDB entry 3BX1(49) and its catalytic triad were used as a query to perform motif search against other PDB entries.^50^ The resulting set was filtered with PISCES^51^ to achieve <95% pairwise sequence identity and aligned to the reference by the side chain of the catalytic His. Lys NZ and Arg CZ atoms served as proxies for the localization of the positive charge, while Asp CG and Glu CD were used to track the location of negative charges. PyMol sessions with the superposed sets are available.

S8A family sequences were retrieved from InterPro (entry IRP015500).^52^ Only reviewed entries were kept. A nonredundant subset was obtained with CD-HIT with 90% identity threshold.^53^ We used MAFFT to construct the sequence alignment.^54^

### Model Preparation

Complex structure was taken from our previous work.^24^ We used amber99sb-ildn force field.^55,56^ Calcium ions were described with COM parameters by Li et al.^57,58^ QM region included side chains of the catalytic triad and residues P1, P2, and P1′ of the peptide VAHAL|AQTVP. Within the peptide, the QM/MM boundary cut through covalent bonds C(QM)-CA(MM) and N(QM)-CA(MM), and QM atoms were capped by hydrogen link atoms. The Amber charge correction scheme was used.^24^ For the deacylation stage, a water molecule was added. The QM region was modeled with Gromacs-DFTBplus interface,^59^ DFTB3 method^60^ with 3ob-3–1 parameters^61^ and D3H5 dispersion and h-bond corrections^62^ similar to what we used before.^63,64^ We used periodic boundary conditions and full PME treatment of the electrostatic interactions. Temperature was controlled with the velocity rescale thermostat at 300 K.^65^

To construct charge-shifted systems, we first assigned backbone partial charges according to the neutral form of the residue from the force field. Charges on side chain atoms were divided by 2 and then shifted to achieve net zero charge for the whole residue (Tables S1, S2, and S4). This was done to create a version of the residue that can still be considered polar and does not have overly unrealistic charges on the aliphatic groups. To evaluate the potential impact of such a choice, we created additional “zeroed” systems. For them, side chain charges were divided by a factor needed to bring the net charge to zero. Since it almost effectively zeroed side chain charges, this version represented a substitution to the apolar residue, while the original represented a substitution to the uncharged polar one. Quantitative results reported for “polar” versions in the paper were found to be reproduced and elevated when the “apolar” scheme was applied (Tables S8–S10).

To keep all systems identical except for the charge, 10 kcal/mol/Å^2^ positional restraints were applied to all backbone atoms more than 7 Å from the center of the imidazole ring of the catalytic His64. We used the same reference configuration for positional restraints for all systems. Additionally, distance restraints were applied to keep the reference Asp60 interaction profile when its charges were rescaled. They consisted of five upper wall restraints along the hydrogen bonds, with starting distance equal to 90th percentile of the unbiased QM/MM MD distribution across all reaction stages (Figure S8). The force constant was set to 20 kcal/mol/Å^2^.

To equilibrate classic systems, we used the same multistep scheme as before.^11^ Afterward, 5 replicas 500 ns each were sampled. For the K94M system, we sampled 1.5 μs per replica.

### QM/MM Simulations and Reaction Sampling

Initial models of 6 stable reaction states were built based on our previous work.^24^ They were then minimized with steepest descent and subjected to 10 ps NVT QM/MM MD with a 1 fs time step. Charge modifications to topologies were applied only afterward. For unmodified models, for each reaction state, we performed an additional 20 ps of sampling in 8 replicas. Last 10 ps were used to populate training sets for DeepTDA variables.^66^ Separate variables were trained for the acylation and deacylation stages.

The input vector for the acylation stage consisted of all pairwise distances between 9 atoms normalized with a switch function (PLUMED input files are provided). The atom set included Ser221 atoms OG and HG; His64 NE2; P1 atoms C, O, and CA; P1′ atoms N, H, and CA (Figure S9A). We used all atoms that change their bonding or can define out-of-plane movements for substrate’s C and N atoms. For the deacylation stage, instead of P1′ atoms we used OW and HW atoms of the water molecule (8 atoms in total, Figure S9B). Sampled distributions of all input distances are provided (Figures S10 and S11). The mlcolvar library was used to train DeepTDA variables.^67^ Centers of distributions were set at −10, 0, 10, with associated widths of 0.4, 0.3, 0.4 for both stages. We used [36, 72, 36, 1] neurons per layer for the acylation stage, and [28, 56, 28, 1] for the deacylation stage. Models converged to validation loss <0.02 in 152 and 232 epochs, respectively.

Since neural networks are known to extrapolate poorly, we ensured that our production simulations sample within the convex hull of the provided distance distributions. We applied wall potentials with force constants of 20 kcal/mol/Å^2^ on distances below 0.5% and above 99.5% percentiles of the training set distributions. Additional potentials were applied to prevent the inversion of the NH_2_ group of the product and dissociation of the peptide (see the provided PLUMED input files).

We used Plumed v2.9.0 for enhanced sampling.^68^ We sampled reactions with OPES explore^69^ running with 8 parallel walkers for 100 ps with 0.5 fs time steps. Each simulation was performed in 5 replicates. Potential was updated every 200 steps, and the barrier parameter was set to 80 kJ/mol. We additionally used a SIGMA_MIN option at 0.3 CV units. To construct free energy profiles, we discarded the first 5 ps of sampling based on the stabilization behavior of the rct factor.

## Conclusions

In this study, we successfully devised and applied a molecular modeling approach to decouple local and nonlocal influences of conserved charged residues that surround active sites of enzymes but are not in direct contact with them. It allowed us to identify a pair of evolved residues in the structure of subtilisin Carlsberg, whose opposite charges have opposite effects on the reaction efficiency. The discovery of Asp60, contributing more than 2 kcal/mol to lowering the activation barrier, enforces the potential of rationally engineering electrostatic fields in *de novo* enzyme design. The conserved positive charge of Lys94 hindered the reaction, which is an intriguing case of evolutionary trade-offs observed for modern natural enzymes. Such insights inform how natural enzymatic activities could be surpassed via the incorporation of noncanonical amino acids. In addition, the developed data-driven collective variables may be used to power efficient reaction sampling for similar enzymes and pave the way for computational enzyme activity assays.

## Data Availability

PyMOL sessions with conserved charge locations, sequence alignment, DeepTDA models, and PLUMED input files are deposited to Zenodo: 10.5281/zenodo.13894221.

## Supporting Information Available

Tables S1, S2, S4: rescaled charges utilized to model the “neutral” version of charged residues; Tables S3, S5: calculated free energy parameters of the reaction catalyzed by subtilisin Carlsberg with “neutral” model of Glu54 and Asp60 without h-bonding restraints; Table S6: amino acids at position 54 in S8A family enzymes; Table S7: selected measurements of the most probable TS structures; Tables S8–S10: calculated free energy parameters of the reaction catalyzed by subtilisin Carlsberg with different ways to delete a charge; Figure S1: profile reconstruction errors with deepTDA-powered reaction sampling; Figure S2: charge rescaling on Asp60 or Lys94 does not lead to pronounced unfolding, conformational change, or substrate unbinding in 500 ns; Figures S3, S4: dynamical stability of the overall protein structure over the course of 500 ns; Figures S5, S6: dynamical stability of the loop 50–64 over the course of 500 ns; Figure S7: convergence and reproducibility of reaction sampling free energy profiles; Figure S8: distributions of Asp60 hydrogen bond distances in unbiased QM/MM MD minima sampling; Figure S9: atoms used in defining the input layer of DeepTDA variables; Figures S10, S11: distributions of all input layer variables used to construct DeepTDA CVs (PDF)

## References

[ref1] Baker D. An Exciting but Challenging Road Ahead for Computational Enzyme Design. Protein Sci. 2010, 19 (10), 1817–1819. 10.1002/pro.481. 20717908 PMC2998717

[ref2] Kalvet I.; Ortmayer M.; Zhao J.; Crawshaw R.; Ennist N. M.; Levy C.; Roy A.; Green A. P.; Baker D. Design of Heme Enzymes with a Tunable Substrate Binding Pocket Adjacent to an Open Metal Coordination Site. J. Am. Chem. Soc. 2023, 145 (26), 14307–14315. 10.1021/jacs.3c02742. 37341421 PMC10326885

[ref3] Lauko A.; Pellock S. J.; Anischanka I.; Sumida K. H.; Juergens D.; Ahern W.; Shida A.; Hunt A.; Kalvet I.; Norn C.; Humphreys I. R.; Jamieson C.; Kang A.; Brackenbrough E.; Bera A. K.; Sankaran B.; Houk K. N.; Baker D., Computational Design of Serine Hydrolases, bioRxiv, 2024. 10.1101/2024.08.29.610411. PMC12288761 39946508

[ref4] Chaturvedi S. S.; Bím D.; Christov C. Z.; Alexandrova A. N. From Random to Rational: Improving Enzyme Design through Electric Fields, Second Coordination Sphere Interactions, and Conformational Dynamics. Chem. Sci. 2023, 14 (40), 10997–11011. 10.1039/D3SC02982D. 37860658 PMC10583697

[ref5] Hansen A. L.; Theisen F. F.; Crehuet R.; Marcos E.; Aghajari N.; Willemoës M. Carving out a Glycoside Hydrolase Active Site for Incorporation into a New Protein Scaffold Using Deep Network Hallucination. ACS Synth. Biol. 2024, 13 (3), 862–875. 10.1021/acssynbio.3c00674. 38357862 PMC10949244

[ref6] Risso V. A.; Romero-Rivera A.; Gutierrez-Rus L. I.; Ortega-Muñoz M.; Santoyo-Gonzalez F.; Gavira J. A.; Sanchez-Ruiz J. M.; Kamerlin S. C. L. Enhancing a Enzyme Activity by Computationally-Focused Ultra-Low-Throughput Screening. Chem. Sci. 2020, 11 (24), 6134–6148. 10.1039/D0SC01935F. 32832059 PMC7407621

[ref7] Vidossich P.; Castañeda Moreno L. E.; Mota C.; de Sanctis D.; Miscione G. P.; De Vivo M. Functional Implications of Second-Shell Basic Residues for dUTPase DR2231 Enzymatic Specificity. ACS Catal. 2020, 10 (23), 13825–13833. 10.1021/acscatal.0c04148.

[ref8] Genna V.; Colombo M.; De Vivo M.; Marcia M. Second-Shell Basic Residues Expand the Two-Metal-Ion Architecture of DNA and RNA Processing Enzymes. Structure 2018, 26 (1), 40–50.e2. 10.1016/j.str.2017.11.008. 29225080 PMC5758106

[ref9] Deng J.; Cui Q. Second-Shell Residues Contribute to Catalysis by Predominately Preorganizing the Apo State in PafA. J. Am. Chem. Soc. 2023, 145 (20), 11333–11347. 10.1021/jacs.3c02423. 37172218 PMC10810092

[ref10] Schwartz S. D. Protein Dynamics and Enzymatic Catalysis. J. Phys. Chem. B 2023, 127 (12), 2649–2660. 10.1021/acs.jpcb.3c00477. 36944023 PMC10072970

[ref11] Zlobin A.; Smirnov I.; Golovin A. Dynamic Interchange between Two Protonation States Is Characteristic of Active Sites of Cholinesterases. Protein Sci. 2024, 33 (8), e5100 10.1002/pro.5100. 39022909 PMC11255601

[ref12] Ruiz-Pernía J. J.; Świderek K.; Bertran J.; Moliner V.; Tuñón I. Electrostatics as a Guiding Principle in Understanding and Designing Enzymes. J. Chem. Theory Comput. 2024, 20 (5), 1783–1795. 10.1021/acs.jctc.3c01395. 38410913 PMC10938506

[ref13] Jindal G.; Warshel A. Misunderstanding the Preorganization Concept Can Lead to Confusions about the Origin of Enzyme Catalysis. Proteins 2017, 85 (12), 2157–2161. 10.1002/prot.25381. 28905418 PMC5760166

[ref14] Jabeen H.; Beer M.; Spencer J.; van der Kamp M. W.; Bunzel H. A.; Mulholland A. J. Electric Fields Are a Key Determinant of Carbapenemase Activity in Class A β-Lactamases. ACS Catal. 2024, 14 (9), 7166–7172. 10.1021/acscatal.3c05302. 38721371 PMC11075022

[ref15] Siddiqui S. A.; Stuyver T.; Shaik S.; Dubey K. D. Designed Local Electric Fields-Promising Tools for Enzyme Engineering. JACS Au 2023, 3 (12), 3259–3269. 10.1021/jacsau.3c00536. 38155642 PMC10752214

[ref16] Świderek K.; Tuñón I.; Moliner V.; Bertran J. Protein Flexibility and Preorganization in the Design of Enzymes. The Kemp Elimination Catalyzed by HG3.17. ACS Catal. 2015, 5 (4), 2587–2595. 10.1021/cs501904w.

[ref17] Welborn V. V.; Ruiz Pestana L.; Head-Gordon T. Computational Optimization of Electric Fields for Better Catalysis Design. Nat. Catal. 2018, 1 (9), 649–655. 10.1038/s41929-018-0109-2.

[ref18] Birch-Price Z.; Hardy F. J.; Lister T. M.; Kohn A. R.; Green A. P. Noncanonical Amino Acids in Biocatalysis. Chem. Rev. 2024, 124 (14), 8740–8786. 10.1021/acs.chemrev.4c00120. 38959423 PMC11273360

[ref19] Ding W.; Yu W.; Chen Y.; Lao L.; Fang Y.; Fang C.; Zhao H.; Yang B.; Lin S. Rare Codon Recoding for Efficient Noncanonical Amino Acid Incorporation in Mammalian Cells. Science 2024, 384 (6700), 1134–1142. 10.1126/science.adm8143. 38843324

[ref20] Birch-Price Z.; Taylor C. J.; Ortmayer M.; Green A. P. Engineering Enzyme Activity Using an Expanded Amino Acid Alphabet. Protein Eng., Des. Sel. 2023, 36, gzac013 10.1093/protein/gzac013. 36370045 PMC9863031

[ref21] Tournier V.; Duquesne S.; Guillamot F.; Cramail H.; Taton D.; Marty A.; André I. Enzymes Power for Plastics Degradation. Chem. Rev. 2023, 123 (9), 5612–5701. 10.1021/acs.chemrev.2c00644. 36916764

[ref22] David Troncoso F.; Alberto Sánchez D.; Luján Ferreira M. Production of Plant Proteases and New Biotechnological Applications: An Updated Review. ChemistryOpen 2022, 11 (3), e202200017 10.1002/open.202200017. 35286022 PMC8919702

[ref23] Bussi G.; Laio A. Using Metadynamics to Explore Complex Free-Energy Landscapes. Nat. Rev. Phys. 2020, 2 (4), 200–212. 10.1038/s42254-020-0153-0.

[ref24] Zlobin A.; Belyaeva J.; Golovin A. Challenges in Protein QM/MM Simulations with Intra-Backbone Link Atoms. J. Chem. Inf. Model. 2023, 63 (2), 546–560. 10.1021/acs.jcim.2c01071. 36633836

[ref25] Díaz-Cervantes E.; Robles J.; Solà M.; Swart M. The Peptide Bond Rupture Mechanism in the Serine Proteases: An Study Based on Sequential Scale Models. Phys. Chem. Chem. Phys. 2023, 25 (11), 8043–8049. 10.1039/D2CP04872H. 36876585

[ref26] Lima M. C. P.; Seabra G. M. Reaction Mechanism of the Dengue Virus Serine Protease: A QM/MM Study. Phys. Chem. Chem. Phys. 2016, 18 (44), 30288–30296. 10.1039/C6CP03209E. 27341353

[ref27] Makarov M.; Sanchez Rocha A. C.; Krystufek R.; Cherepashuk I.; Dzmitruk V.; Charnavets T.; Faustino A. M.; Lebl M.; Fujishima K.; Fried S. D.; Hlouchova K. Early Selection of the Amino Acid Alphabet Was Adaptively Shaped by Biophysical Constraints of Foldability. J. Am. Chem. Soc. 2023, 145 (9), 5320–5329. 10.1021/jacs.2c12987. 36826345 PMC10017022

[ref28] Shandell M. A.; Tan Z.; Cornish V. W. Genetic Code Expansion: A Brief History and Perspective. Biochemistry 2021, 60 (46), 3455–3469. 10.1021/acs.biochem.1c00286. 34196546 PMC8613843

[ref29] Costello A.; Peterson A. A.; Lanster D. L.; Li Z.; Carver G. D.; Badran A. H. Efficient Genetic Code Expansion without Host Genome Modifications, Nat. Biotechnol., 2024. 10.1038/s41587-024-02385-y. 39261591

[ref30] Kwok H. S.; Vargas-Rodriguez O.; Melnikov S. V.; Söll D. Engineered Aminoacyl-tRNA Synthetases with Improved Selectivity toward Noncanonical Amino Acids. ACS Chem. Biol. 2019, 14 (4), 603–612. 10.1021/acschembio.9b00088. 30933556 PMC6642615

[ref31] Dunkelmann D. L.; Piedrafita C.; Dickson A.; Liu K. C.; Elliott T. S.; Fiedler M.; Bellini D.; Zhou A.; Cervettini D.; Chin J. W. Adding α,α-Disubstituted and β-Linked Monomers to the Genetic Code of an Organism. Nature 2024, 625 (7995), 603–610. 10.1038/s41586-023-06897-6. 38200312 PMC10794150

[ref32] Nicoll C. R.; Massari M.; Fraaije M. W.; Mascotti M. L.; Mattevi A. Impact of Ancestral Sequence Reconstruction on Mechanistic and Structural Enzymology. Curr. Opin. Struct. Biol. 2023, 82, 102669 10.1016/j.sbi.2023.102669. 37544113

[ref33] Bradshaw R. T.; Dziedzic J.; Skylaris C.-K.; Essex J. W. The Role of Electrostatics in Enzymes: Do Biomolecular Force Fields Reflect Protein Electric Fields?. J. Chem. Inf. Model. 2020, 60 (6), 3131–3144. 10.1021/acs.jcim.0c00217. 32298113

[ref34] Yan S.; Ji X.; Peng W.; Wang B. Evaluating the Transition State Stabilization/Destabilization Effects of the Electric Fields from Scaffold Residues by a QM/MM Approach. J. Phys. Chem. B 2023, 127 (19), 4245–4253. 10.1021/acs.jpcb.3c01054. 37155960

[ref35] Peng W.; Yan S.; Zhang X.; Liao L.; Zhang J.; Shaik S.; Wang B. How Do Preorganized Electric Fields Function in Catalytic Cycles? The Case of the Enzyme Tyrosine Hydroxylase. J. Am. Chem. Soc. 2022, 144 (44), 20484–20494. 10.1021/jacs.2c09263. 36282048

[ref36] Zlobin A.; Ermidis A.-P.; Maslova V.; Belyaeva J.; Golovin A. Exploiting Structural Constraints of Proteolytic Catalytic Triads for Fast Supercomputer Scaffold Probing in Enzyme Design Studies. In Communications in Computer and Information Science, Voevodin V.; Sobolev S., Eds.; Springer International Publishing: Cham, 2021; pp. 58–72.

[ref37] Beker W.; Sokalski W. A. Bottom-Up Nonempirical Approach To Reducing Search Space in Enzyme Design Guided by Catalytic Fields. J. Chem. Theory Comput. 2020, 16 (5), 3420–3429. 10.1021/acs.jctc.0c00139. 32282205 PMC7467639

[ref38] Raucci U.; Rizzi V.; Parrinello M. Discover, Sample, and Refine: Exploring Chemistry with Enhanced Sampling Techniques. J. Phys. Chem. Lett. 2022, 13 (6), 1424–1430. 10.1021/acs.jpclett.1c03993. 35119863

[ref39] Smith Z.; Ravindra P.; Wang Y.; Cooley R.; Tiwary P. Discovering Protein Conformational Flexibility through Artificial-Intelligence-Aided Molecular Dynamics. J. Phys. Chem. B 2020, 124 (38), 8221–8229. 10.1021/acs.jpcb.0c03985. 32841026

[ref40] Elishav O.; Podgaetsky R.; Meikler O.; Hirshberg B. Collective Variables for Conformational Polymorphism in Molecular Crystals. J. Phys. Chem. Lett. 2023, 14 (4), 971–976. 10.1021/acs.jpclett.2c03491. 36689770 PMC9900638

[ref41] Zou Z.; Tiwary P. Enhanced Sampling of Crystal Nucleation with Graph Representation Learnt Variables. J. Phys. Chem. B 2024, 128 (12), 3037–3045. 10.1021/acs.jpcb.4c00080. 38502931

[ref42] Ansari N.; Rizzi V.; Parrinello M. Water Regulates the Residence Time of Benzamidine in Trypsin. Nat. Commun. 2022, 13 (1), 5438 10.1038/s41467-022-33104-3. 36114175 PMC9481606

[ref43] Wang Y.; Parmar S.; Schneekloth J. S.; Tiwary P. Interrogating RNA-Small Molecule Interactions with Structure Probing and Artificial Intelligence-Augmented Molecular Simulations. ACS Cent. Sci. 2022, 8 (6), 741–748. 10.1021/acscentsci.2c00149. 35756372 PMC9228567

[ref44] Mehdi S.; Wang D.; Pant S.; Tiwary P. Accelerating All-Atom Simulations and Gaining Mechanistic Understanding of Biophysical Systems through State Predictive Information Bottleneck. J. Chem. Theory Comput. 2022, 18 (5), 3231–3238. 10.1021/acs.jctc.2c00058. 35384668 PMC9297332

[ref45] France-Lanord A.; Vroylandt H.; Salanne M.; Rotenberg B.; Saitta A. M.; Pietrucci F. Data-Driven Path Collective Variables. J. Chem. Theory Comput. 2024, 20 (8), 3069–3084. 10.1021/acs.jctc.4c00123. 38619076

[ref46] Fröhlking T.; Bonati L.; Rizzi V.; Gervasio F. L. Deep Learning Path-like Collective Variable for Enhanced Sampling Molecular Dynamics. J. Chem. Phys. 2024, 160 (17), 174109 10.1063/5.0202156. 38748013

[ref47] McSloy A.; Fan G.; Sun W.; Hölzer C.; Friede M.; Ehlert S.; Schütte N.-E.; Grimme S.; Frauenheim T.; Aradi B. TBMaLT, a Flexible Toolkit for Combining Tight-Binding and Machine Learning. J. Chem. Phys. 2023, 158 (3), 034801 10.1063/5.0132892. 36681630

[ref48] Käser S.; Vazquez-Salazar L. I.; Meuwly M.; Töpfer K. Neural Network Potentials for Chemistry: Concepts, Applications and Prospects. Digit Discov. 2023, 2 (1), 28–58. 10.1039/D2DD00102K. 36798879 PMC9923808

[ref49] Micheelsen P. O.; Vévodová J.; De Maria L.; Ostergaard P. R.; Friis E. P.; Wilson K.; Skjøt M. Structural and Mutational Analyses of the Interaction between the Barley Alpha-Amylase/subtilisin Inhibitor and the Subtilisin Savinase Reveal a Novel Mode of Inhibition. J. Mol. Biol. 2008, 380 (4), 681–690. 10.1016/j.jmb.2008.05.034. 18556023

[ref50] Bittrich S.; Burley S. K.; Rose A. S. Real-Time Structural Motif Searching in Proteins Using an Inverted Index Strategy. PLoS Comput. Biol. 2020, 16 (12), e1008502 10.1371/journal.pcbi.1008502. 33284792 PMC7746303

[ref51] Wang G.; Dunbrack R. L. Jr PISCES: A Protein Sequence Culling Server. Bioinformatics 2003, 19 (12), 1589–1591. 10.1093/bioinformatics/btg224. 12912846

[ref52] Paysan-Lafosse T.; Blum M.; Chuguransky S.; Grego T.; Pinto B. L.; Salazar G. A.; Bileschi M. L.; Bork P.; Bridge A.; Colwell L.; Gough J.; Haft D. H.; Letunić I.; Marchler-Bauer A.; Mi H.; Natale D. A.; Orengo C. A.; Pandurangan A. P.; Rivoire C.; Sigrist C. J. A.; Sillitoe I.; Thanki N.; Thomas P. D.; Tosatto S. C. E.; Wu C. H.; Bateman A. InterPro in 2022. Nucleic Acids Res. 2023, 51 (D1), D418–D427. 10.1093/nar/gkac993. 36350672 PMC9825450

[ref53] Fu L.; Niu B.; Zhu Z.; Wu S.; Li W. CD-HIT: Accelerated for Clustering the next-Generation Sequencing Data. Bioinformatics 2012, 28 (23), 3150–3152. 10.1093/bioinformatics/bts565. 23060610 PMC3516142

[ref54] Madeira F.; Madhusoodanan N.; Lee J.; Eusebi A.; Niewielska A.; Tivey A. R. N.; Lopez R.; Butcher S. The EMBL-EBI Job Dispatcher Sequence Analysis Tools Framework in 2024. Nucleic Acids Res. 2024, 52 (W1), W521–W525. 10.1093/nar/gkae241. 38597606 PMC11223882

[ref55] Belyaeva J.; Zlobin A.; Maslova V.; Golovin A. Modern Non-Polarizable Force Fields Diverge in Modeling the Enzyme-Substrate Complex of a Canonical Serine Protease. Phys. Chem. Chem. Phys. 2023, 25 (8), 6352–6361. 10.1039/D2CP05502C. 36779321

[ref56] Lindorff-Larsen K.; Piana S.; Palmo K.; Maragakis P.; Klepeis J. L.; Dror R. O.; Shaw D. E. Improved Side-Chain Torsion Potentials for the Amber ff99SB Protein Force Field. Proteins 2010, 78 (8), 1950–1958. 10.1002/prot.22711. 20408171 PMC2970904

[ref57] Zlobin A.; Diankin I.; Pushkarev S.; Golovin A. Probing the Suitability of Different Ca Parameters for Long Simulations of Diisopropyl Fluorophosphatase. Molecules 2021, 26 (19), 5839 10.3390/molecules26195839. 34641383 PMC8510429

[ref58] Li Z.; Song L. F.; Li P.; Merz K. M. Jr Systematic Parametrization of Divalent Metal Ions for the OPC3, OPC, TIP3P-FB, and TIP4P-FB Water Models. J. Chem. Theory Comput. 2020, 16 (7), 4429–4442. 10.1021/acs.jctc.0c00194. 32510956 PMC8173364

[ref59] Gillet N.; Elstner M.; Kubař T. Coupled-Perturbed DFTB-QM/MM Metadynamics: Application to Proton-Coupled Electron Transfer. J. Chem. Phys. 2018, 149 (7), 072328 10.1063/1.5027100. 30134697

[ref60] Gaus M.; Cui Q.; Elstner M. DFTB3: Extension of the Self-Consistent-Charge Density-Functional Tight-Binding Method (SCC-DFTB). J. Chem. Theory Comput. 2011, 7 (4), 931–948. 10.1021/ct100684s. PMC3509502 23204947

[ref61] Gaus M.; Goez A.; Elstner M. Parametrization and Benchmark of DFTB3 for Organic Molecules. J. Chem. Theory Comput. 2013, 9 (1), 338–354. 10.1021/ct300849w. 26589037

[ref62] ŘezáČ J. Empirical Self-Consistent Correction for the Description of Hydrogen Bonds in DFTB3. J. Chem. Theory Comput. 2017, 13 (10), 4804–4817. 10.1021/acs.jctc.7b00629. 28949517

[ref63] Zlobin A. S.; Zalevsky A. O.; Mokrushina Y. A.; Kartseva O. V.; Golovin A. V.; Smirnov I. V. The Preferable Binding Pose of Canonical Butyrylcholinesterase Substrates Is Unproductive for Echothiophate. Acta Nat. 2018, 10 (4), 121–124. 10.32607/20758251-2018-10-4-121-124. PMC6351040 30713771

[ref64] Zlobin A.; Golovin A. Between Protein Fold and Nucleophile Identity: Multiscale Modeling of the TEV Protease Enzyme-Substrate Complex. ACS Omega 2022, 7 (44), 40279–40292. 10.1021/acsomega.2c05201. 36385818 PMC9647873

[ref65] Bussi G.; Donadio D.; Parrinello M. Canonical Sampling through Velocity Rescaling. J. Chem. Phys. 2007, 126 (1), 014101 10.1063/1.2408420. 17212484

[ref66] Trizio E.; Parrinello M. From Enhanced Sampling to Reaction Profiles. J. Phys. Chem. Lett. 2021, 12 (35), 8621–8626. 10.1021/acs.jpclett.1c02317. 34469175

[ref67] Bonati L.; Trizio E.; Rizzi A.; Parrinello M. A Unified Framework for Machine Learning Collective Variables for Enhanced Sampling Simulations: Mlcolvar. J. Chem. Phys. 2023, 159 (1), 014801 10.1063/5.0156343. 37409767

[ref68] Promoting Transparency and Reproducibility in Enhanced Molecular Simulations. Nat. Methods 2019, 16 (8), 670–673. 10.1038/s41592-019-0506-8. 31363226

[ref69] Invernizzi M.; Parrinello M. Exploration vs Convergence Speed in Adaptive-Bias Enhanced Sampling. J. Chem. Theory Comput. 2022, 18 (6), 3988–3996. 10.1021/acs.jctc.2c00152. 35617155 PMC9202311

