# Supplementary material for: Long-Range Electrostatics in Serine Proteases: Machine Learning-Driven Reaction Sampling Yields Insights for Enzyme Design
Source: J Chem Inf Model. 2025 Feb 10;65(4):2003–13. doi: 10.1021/acs.jcim.4c01827 (PMC11863386; doi:10.1021/acs.jcim.4c01827)
Supplement: Supplementary file 1 — ci4c01827_si_001.pdf [file ci4c01827_si_001.pdf]

Role of Second-Shell Electrostatics in Serine Proteases: Machine Learning-Driven Reaction Sampling Yields Insights for Enzyme Design

Alexander Zlobin[1,2,*], Valentina Maslova[2], Julia Beliaeva[1,2,3], Jens Meiler[1,4,5,6], Andrey Golovin[2,7,8]

1. Institute for Drug Discovery, Leipzig University Medical School, Brüderstraße 34, 04103 Leipzig, Germany
2. Faculty of Bioengineering and Bioinformatics, Lomonosov Moscow State University, Leninskie Gory 1, building 73, 119234 Moscow, Russia
3. Institute for Medical Physics and Biophysics, Leipzig University Medical School, Härtelstr. 16-18, 04107 Leipzig, Germany
4. Department of Chemistry, Vanderbilt University, 1234 Stevenson Center Lane, Nashville, TN 37240, Tennessee, USA
5. Center for Structural Biology, Vanderbilt University, PMB 407917, Nashville, TN 37240-7917, Tennessee, USA
6. Center for Scalable Data Analytics and Artificial Intelligence (ScaDS.AI), 04081 Leipzig, Germany
7. Shemyakin and Ovchinnikov Institute of Bioorganic Chemistry, Russian Academy of Sciences, Miklukho-Maklaya 16/10, 117997 Moscow, Russia
8. Belozersky Institute of Physico-Chemical Biology, Lomonosov Moscow State University, Leninskie Gory 1, building 40, 119992 Moscow, Russia

Correspondence: Alexander Zlobin, aleksandr.zlobin@medizin.uni-leipzig.de

SUPPLEMENTARY INFORMATION

Following information is available:
Tables: S1. Rescaled charges utilized to model the “neutral” version of Asp60. S2. Rescaled charges utilized to model the “neutral” version of Lys94. S3. Calculated free energy parameters of the reaction catalyzed by subtilisin Carlsberg with “neutral” model of Glu54. S4. Rescaled charges utilized to model the “neutral” version of Glu54. S5. Calculated free energy parameters of the reaction catalyzed by subtilisin Carlsberg model with “Neutral” Asp60 without h-bonding restraints. S6. Amino acids at position 54 in S8A family enzymes. S7. Selected measurements of the most probable TS structures. S8. Calculated free energy parameters of the acylation stage of the reaction catalyzed by subtilisin Carlsberg with different ways to delete a charge. S9. Calculated free energy parameters of the deacylation stage of the reaction catalyzed by subtilisin Carlsberg with different ways to delete a charge. S10. Calculated free energy parameters of the reaction catalyzed by subtilisin Carlsberg with “Zeroed” model of Glu54.
Figures: S1. Profile reconstruction errors with deepTDA-powered reaction sampling. S2. Charge rescaling on Asp60 or Lys94 does not lead to pronounced unfolding, conformational change, or substrate unbinding in 500 ns. S3,4. Dynamical stability of the overall protein structure over the course of 500 ns. S5,6. Dynamical stability of the loop 50-64 over the course of 500 ns. S7. Convergence and reproducibility of reaction sampling free energy profiles. S8. Distributions of Asp60 hydrogen bond distances in unbiased QM/MM MD minima sampling. S9. Atoms used in defining the input layer of DeepTDA variables. S10,11. Distributions of all input layer variables used to construct DeepTDA CVs.

Table S1. **Rescaled charges utilized to model the "neutral" version of Asp60.** "Neutral" forms correspond to systems in which partial charges for the sidechain of the respective residue are shifted to yield net zero total charge while remaining polar.

**Atom name**	**Original**	**Rescaled**	**Atom name**	**Original**	**Rescaled**
**N**	-0.5163	-0.4157	**CB**	-0.0303	0.05534167
**H**	0.2936	0.2719	**HB1**	-0.0122	0.06439166
**CA**	0.0381	0.0341	**HB2**	-0.0122	0.06439166
**HA**	0.088	0.0864	**CG**	0.7994	0.47019167
**C**	0.5366	0.5973	**OD1**	-0.8014	-0.33020833
**O**	-0.5819	-0.5679	**OD2**	-0.8014	-0.33020833

Table S2. **Rescaled charges utilized to model the "neutral" version of Lys94.** "Neutral" forms correspond to systems in which partial charges for the sidechain of the respective residue are shifted to yield net zero total charge while remaining polar.

**Atom name**	**Original**	**Rescaled**	**Atom name**	**Original**	**Rescaled**
**N**	-0.3479	-0.4157	**CD**	-0.0479	-0.05056812
**H**	0.2747	0.2719	**HD1**	0.0621	0.00443188
**CA**	-0.24	-0.07206	**HD2**	0.0621	0.00443188
**HA**	0.1426	0.09940	**CE**	-0.0143	-0.03376812
**C**	0.7341	0.5973	**HE1**	0.1135	0.03013188
**O**	-0.5894	-0.5679	**HE2**	0.1135	0.03013188
**CB**	-0.0094	-0.03131813	**NZ**	-0.3854	-0.21931813
**HB1**	0.0362	-0.00851812	**HZ1**	0.34	0.14338188
**HB2**	0.0362	-0.00851812	**HZ2**	0.34	0.14338188
**CG**	0.0187	-0.01726812	**HZ3**	0.34	0.14338188
**HG1**	0.0103	-0.02146812			
**HG2**	0.0103	-0.02146812			

Table S3. **Calculated free energy parameters of the reaction catalyzed by subtilisin Carlsberg with "neutral" model of Glu54.** "Neutral" forms correspond to systems in which partial charges for the sidechain of the respective residue are shifted to yield net zero total charge (Table S4).

	"Neutral" Glu54	
	Acylation stage ΔG, kcal/mol	Deacylation stage ΔG, kcal/mol
RS → TS1	13.8±0.2	11.7±0.1
RS → INT	8.2±0.2	-0.2±0.1
RS → TS2	18.2±0.2	9.1±0.2
RS → PS	1.3±0.1	0.4±0.3

Table S4. **Rescaled charges utilized to model the "neutral" version of Glu54.** "Neutral" forms correspond to systems in which partial charges for the sidechain of the respective residue are shifted to yield net zero total charge while remaining polar.

**Atom name**	**Original**	**Rescaled**	**Atom name**	**Original**	**Rescaled**
**N**	-0.5163	-0.4157	**CB**	0.0560	0.07945556
**H**	0.2936	0.2719	**HB1**	-0.0173	0.04280556
**CA**	0.0397	0.0145	**HB2**	-0.0173	0.04280556
**HA**	0.1105	0.0779	**CG**	0.0136	0.05825556
**C**	0.5366	0.5973	**HG1**	-0.0425	0.03020556
**O**	-0.5819	-0.5679	**HG2**	-0.0425	0.03020556
			**CD**	0.8054	0.45415556
			**OD1**	-0.8188	-0.35794444
			**OD2**	-0.8188	-0.35794444

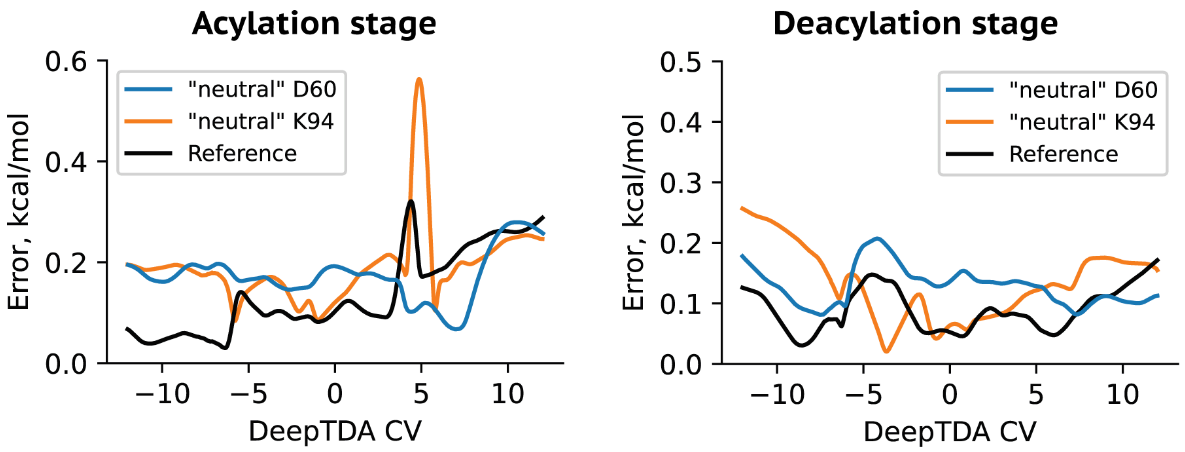

Figure S1. **Profile reconstruction errors with deepTDA-powered reaction sampling.** Shown are values for standard errors of mean calculated for 5 independent simulations.

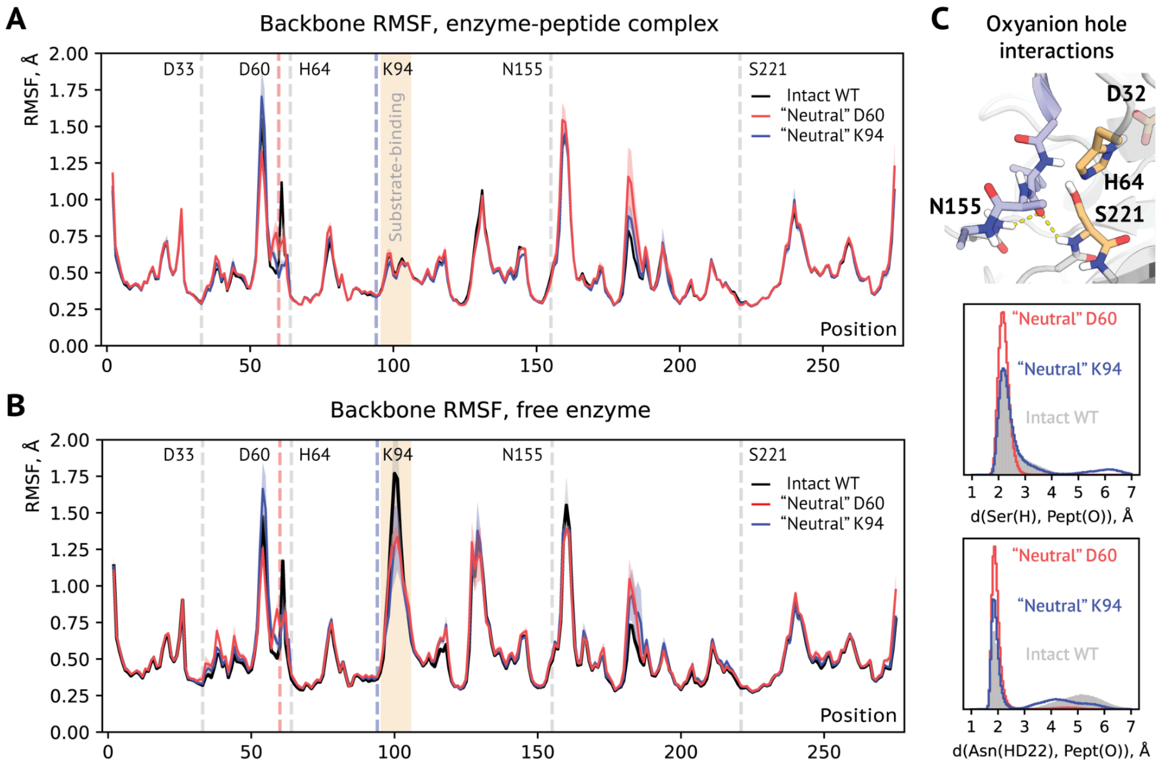

Figure S2. **Charge rescaling on Asp60 or Lys94 does not lead to pronounced unfolding, conformational change, or substrate unbinding in 500 ns. A.** RMSF values for enzyme-peptide complex systems. **B.** RMSF values for free systems. Region 99-103 that forms a beta-sheet with the peptide is highlighted. Dashed lines indicate positions central to the paper's results. **C.** Distribution of distances of two oxyanion hole h-bonds in the last 100 ns of each simulation. Ser(H) corresponds to the S221 backbone H, Asn(HD22) = sidechain HD of Asn155. Non-polar hydrogens are omitted for clarity. Peptide is colored blue, and the catalytic triad is shown in orange.

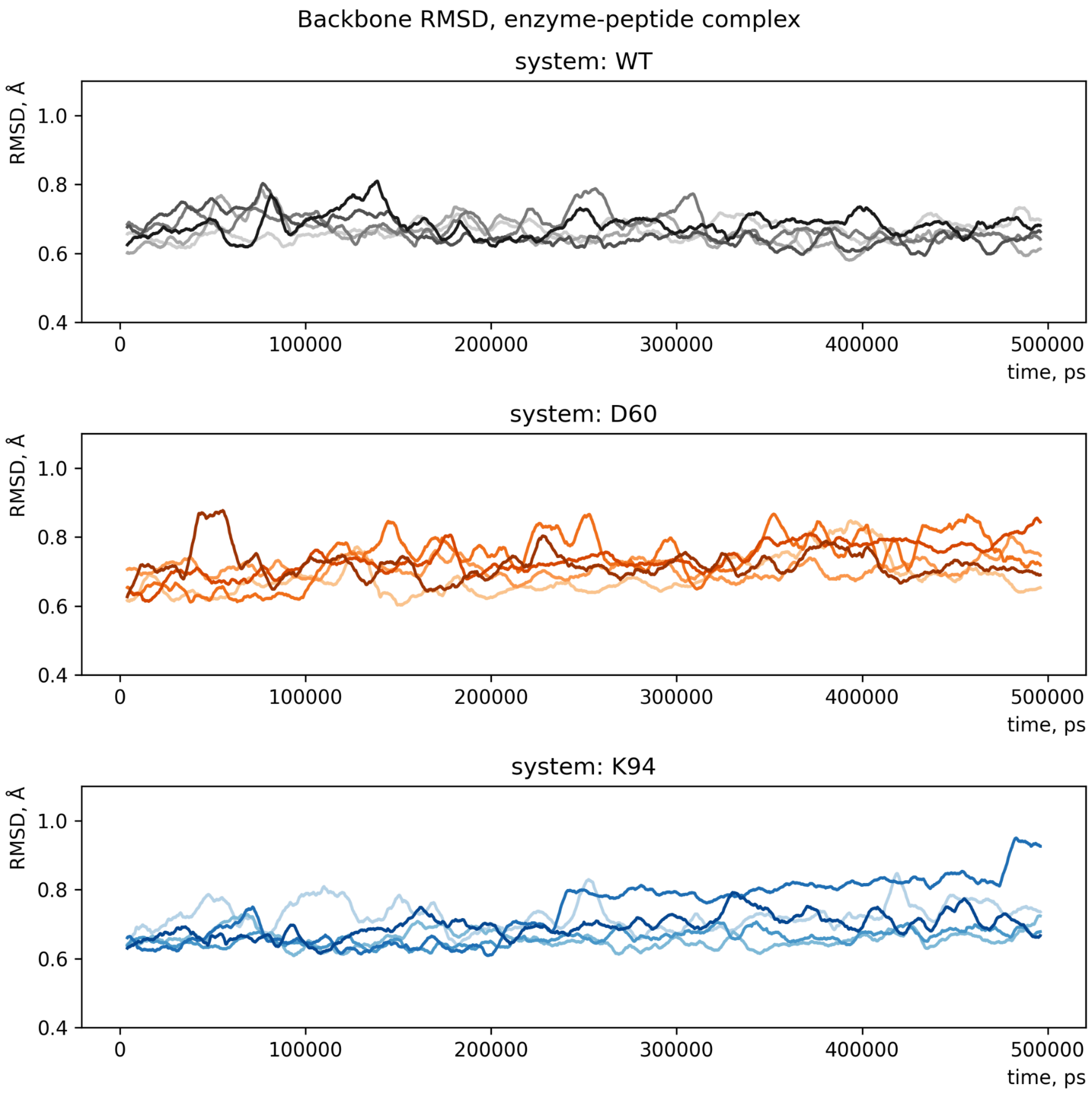

Figure S3. **Dynamical stability of the overall protein structure over the course of 500 ns.** RMSD is calculated after optimal superposition based on protein (without substrate peptide) backbone atoms only.

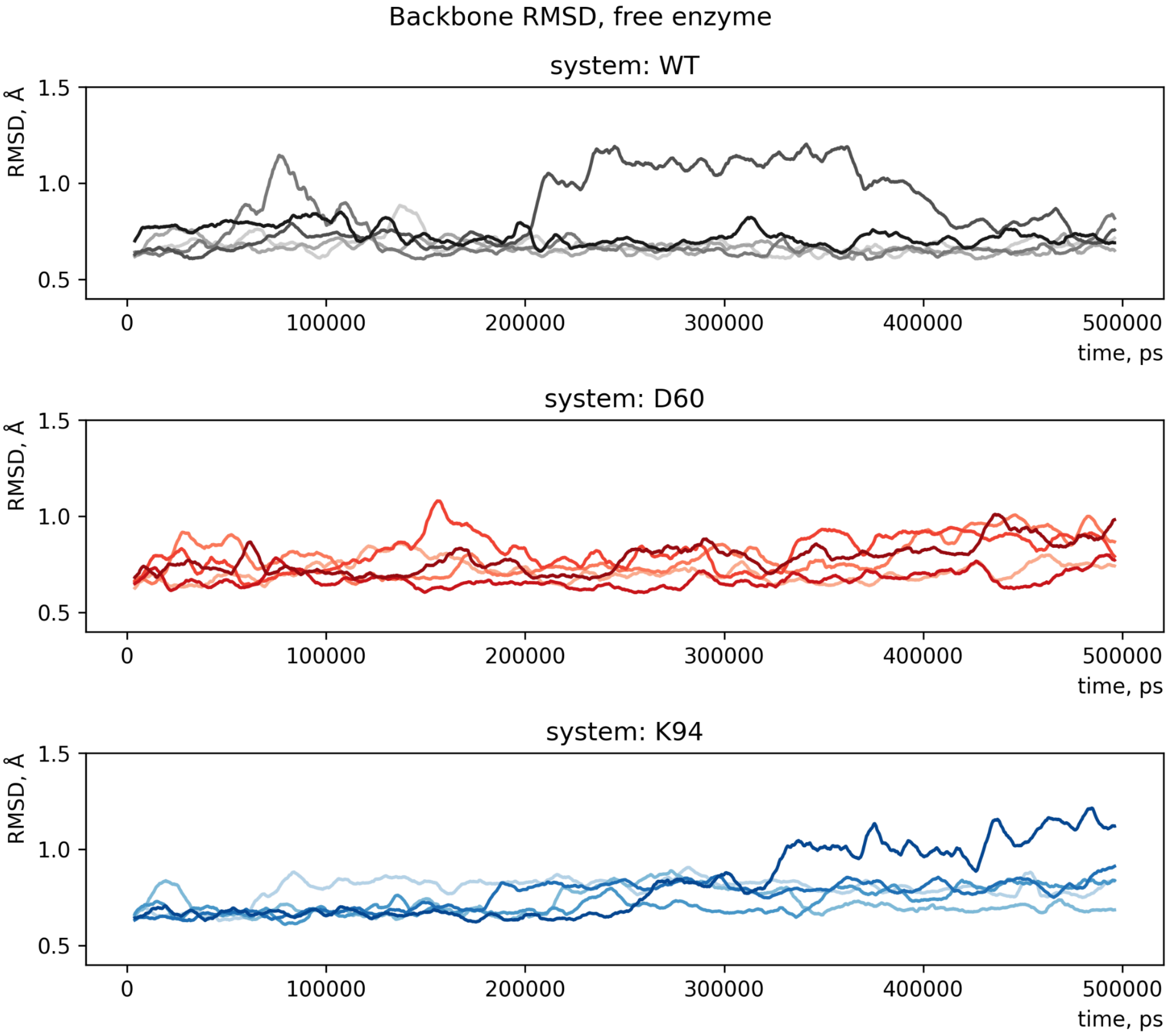

Figure S4. **Dynamical stability of the overall protein structure over the course of 500 ns.** RMSD is calculated after optimal superposition based on backbone atoms only.

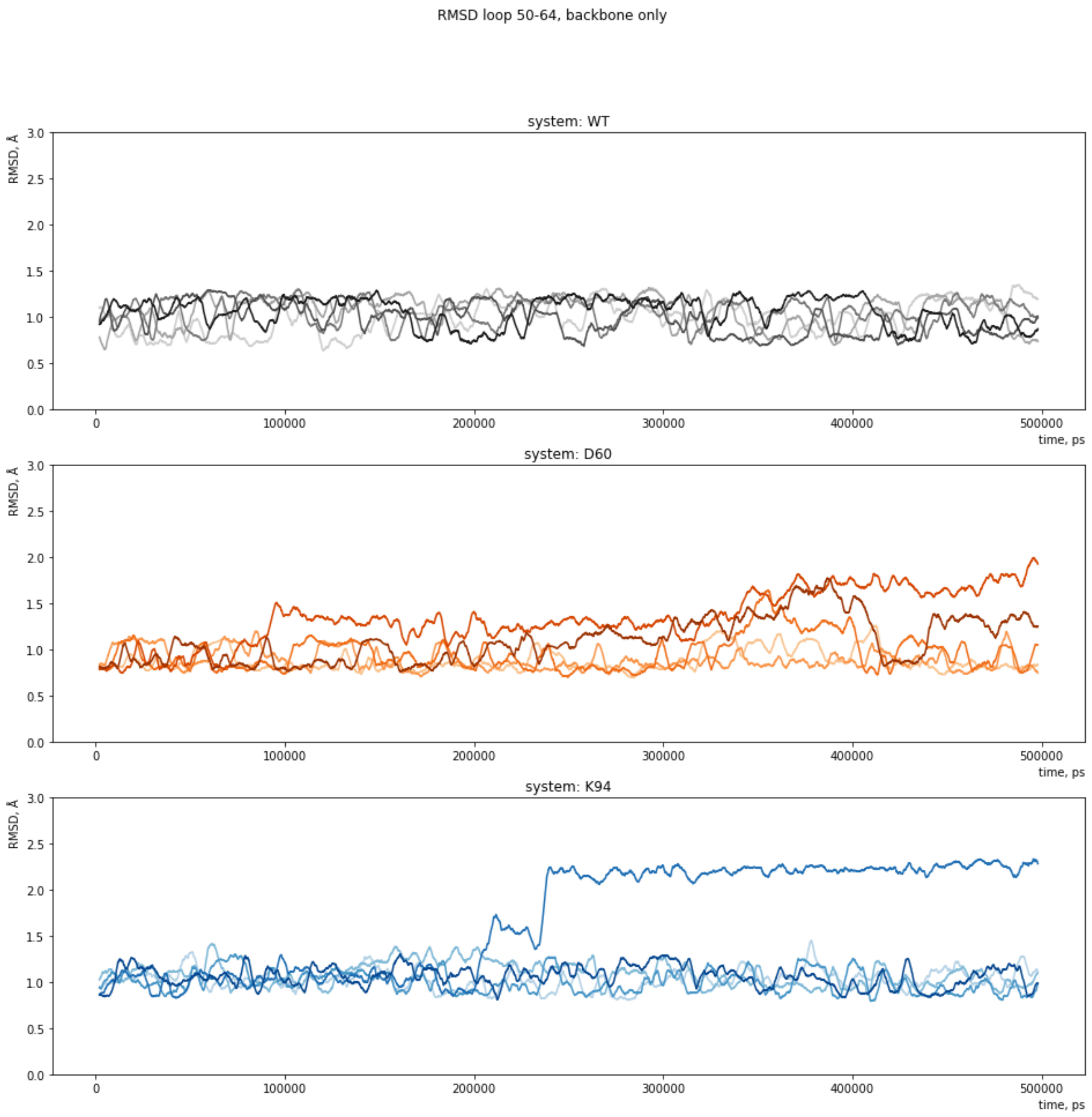

Figure S5. **Dynamical stability of the loop 50-64 over the course of 500 ns.** Values for the enzyme-substrate systems are shown. RMSD is calculated after optimal superposition based on protein (without substrate peptide) backbone atoms only.

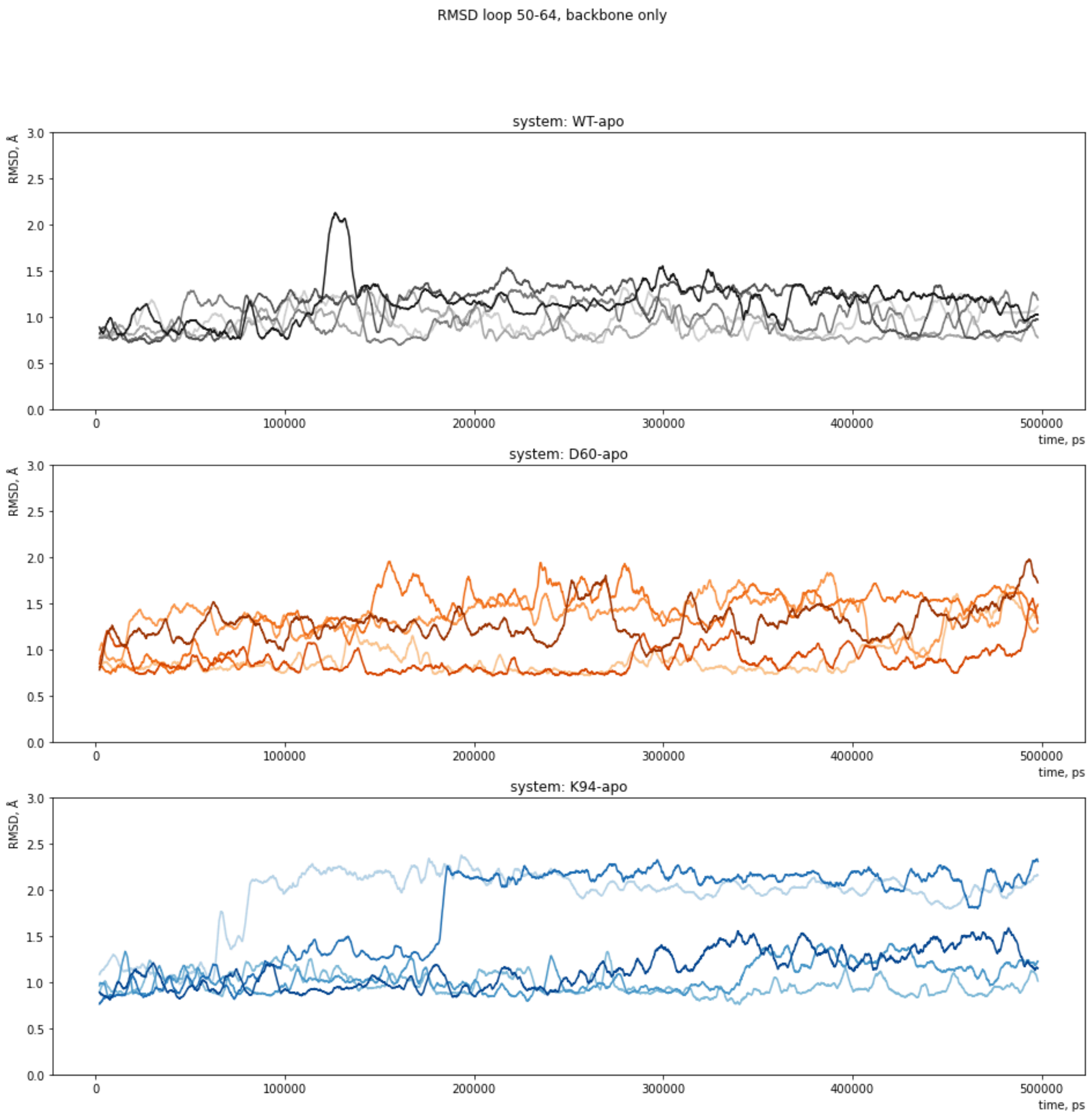

Figure S6. **Dynamical stability of the loop 50-64 over the course of 500 ns.** Values for the free enzyme systems are shown. RMSD is calculated after optimal superposition based on protein backbone atoms only.

Table S5. **Calculated free energy parameters of the reaction catalyzed by subtilisin Carlsberg model with "Neutral" Asp60 without h-bonding restraints.** "Neutral" forms correspond to systems in which partial charges for the sidechain of the corresponding residue are shifted to yield net zero total charge (Table S1 above).

	"Neutral" Asp60 without h-bonding restraints	
	Acylation stage ΔG, kcal/mol	Deacylation stage ΔG, kcal/mol
RS → TS1	14.7±0.1	14.2±0.2
RS → INT	11.4±0.1	2.4±0.2
RS → TS2	20.8±0.2	8.5±0.1
RS → PS	2.1±0.2	-1.2±0.1

Table S6. **Amino acids at position 54 in S8A family enzymes.**

G	25.0%	R	3.9%	L	0.9%
D	13.8%	T	3.9%	C	0.9%
S	12.5%	A	3.4%	M	0.4%
-	9.9%	H	1.3%	V	0.4%
N	9.5%	I	1.3%	Y	0.4%
K	5.6%	Q	0.9%		
E	5.2%	F	0.9%	D+E	19%

Table S7. **Selected measurements of the most probable TS structures.** Values were extracted from the analysis of free energy landscapes. FES are in turn a product of sampling transition state ensembles populated by multiple instances of TS in each walker of each replica.

TS1.1			
Distance, Å	O(Ser) – C(pept)	O(Ser) – H	N(His) – H
WT	2.17	1.44	1.22
D60	2.17	1.46	1.19
K94	2.18	1.43	1.25
TS1.2			
Distance, Å	C(pept) – N(pept)	N(His) – H	N(pept) – H
WT	1.92	1.35	1.49
D60	1.93	1.32	1.49
K94	1.95	1.35	1.49
TS2.1			
Distance, Å	O(water) – C(pept)	O(water) – H	N(His) – H
WT	2.07	1.30	1.40
D60	2.01	1.30	1.35
K94	2.10	1.34	1.44
TS2.2			
Distance, Å	O(Ser) – C(pept)	N(His) – H	O(Ser) – H
WT	2.09	1.36	1.31
D60	2.09	1.32	1.34
K94	2.09	1.38	1.30

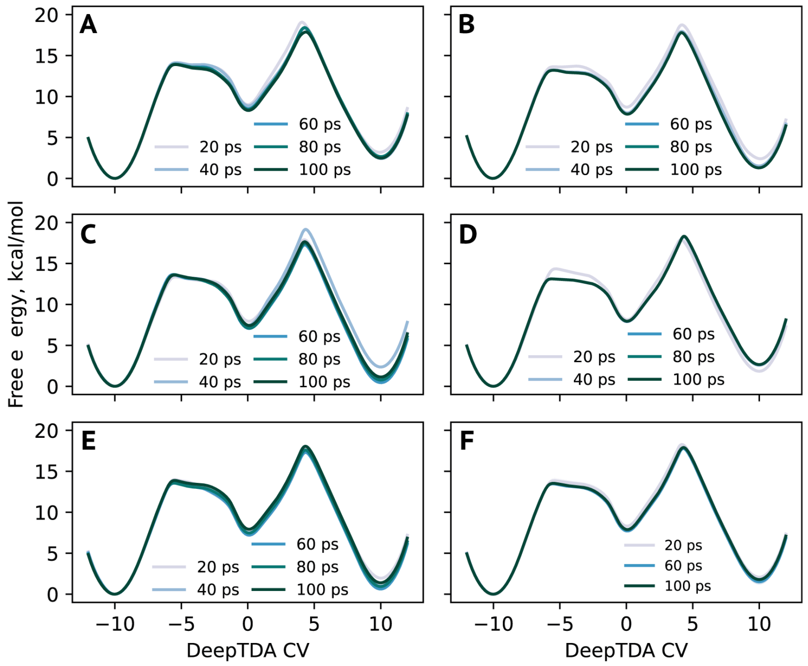

Figure S7. **Convergence and reproducibility of reaction sampling free energy profiles.** Shown is data for the acylation stage for the intact model. **A-E.** Convergence of five individual replicas. **F.** Convergence of the replica average.

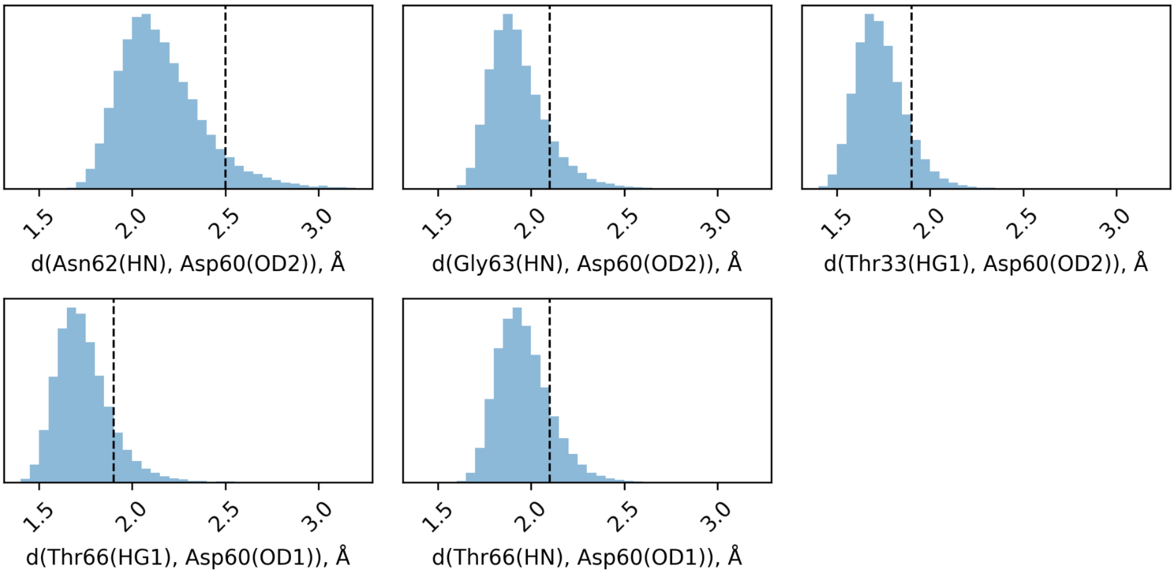

Figure S8. **Distributions of Asp60 hydrogen bond distances in unbiased QM/MM MD minima sampling.** Black dashed line represents the 90th percentile, at which upper walls restraints were placed for Asp60 simulations.

Table S8. **Calculated free energy parameters of the acylation stage of the reaction catalyzed by subtilisin Carlsberg with different ways to delete a charge.** “Neutral” forms correspond to systems in which partial charges for the sidechain of the respective residue are shifted to yield net zero total charge (Tables S1,2,4). For “Zeroed” systems, sidechain charges were divided by a factor needed to bring the net charge to zero.

	Acylation stage				
	Intact	“Neutral” Asp60	“Zeroed” Asp60	“Neutral” Lys94	“Zeroed” Lys94
RS → TS1	13.5±0.1	15.12±0.06	15.9±0.3	13.4±0.1	13.5±0.3
RS → INT	7.9±0.1	10.4±0.1	11.0±0.4	6.8±0.1	7.1±0.3
RS → TS2	17.9±0.1	20.2±0.1	20.5±0.3	17.1±0.1	16.6±0.3
RS → PS	1.8±0.3	1.3±0.4	1.2±0.4	0.9±0.2	1.2±0.5

Table S9. **Calculated free energy parameters of the deacylation stage of the reaction catalyzed by subtilisin Carlsberg with different ways to delete a charge.** “Neutral” forms correspond to systems in which partial charges for the sidechain of the respective residue are shifted to yield net zero total charge (Tables S1,2,4). For “Zeroed” systems, sidechain charges were divided by a factor needed to bring the net charge to zero.

	Deacylation stage				
	Intact	“Neutral” Asp60	“Zeroed” Asp60	“Neutral” Lys94	“Zeroed” Lys94
RS → TS1	11.7±0.2	13.6±0.1	14.5±0.1	11.1±0.1	10.7±0.2
RS → INT	-0.64±0.06	1.4±0.1	3.0±0.1	-1.1±0.3	-1.7±0.2
RS → TS2	8.74±0.04	9.56±0.09	10.6±0.2	9.3±0.2	8.6±0.2
RS → PS	0.33±0.09	-0.2±0.2	0.4±0.2	0.9±0.3	0.1±0.2

Table S10. **Calculated free energy parameters of the reaction catalyzed by subtilisin Carlsberg with “Zeroed” model of Glu54.** For “Zeroed” systems, sidechain charges were divided by a factor needed to bring the net charge to zero.

	“Zeroed” Glu54	
	Acylation stage ΔG, kcal/mol	Deacylation stage ΔG, kcal/mol
RS → TS1	14.0±0.2	11.6±0.1
RS → INT	8.0±0.3	-0.7±0.1
RS → TS2	17.7±0.1	8.8±0.1
RS → PS	1.0±0.2	0.06±0.2

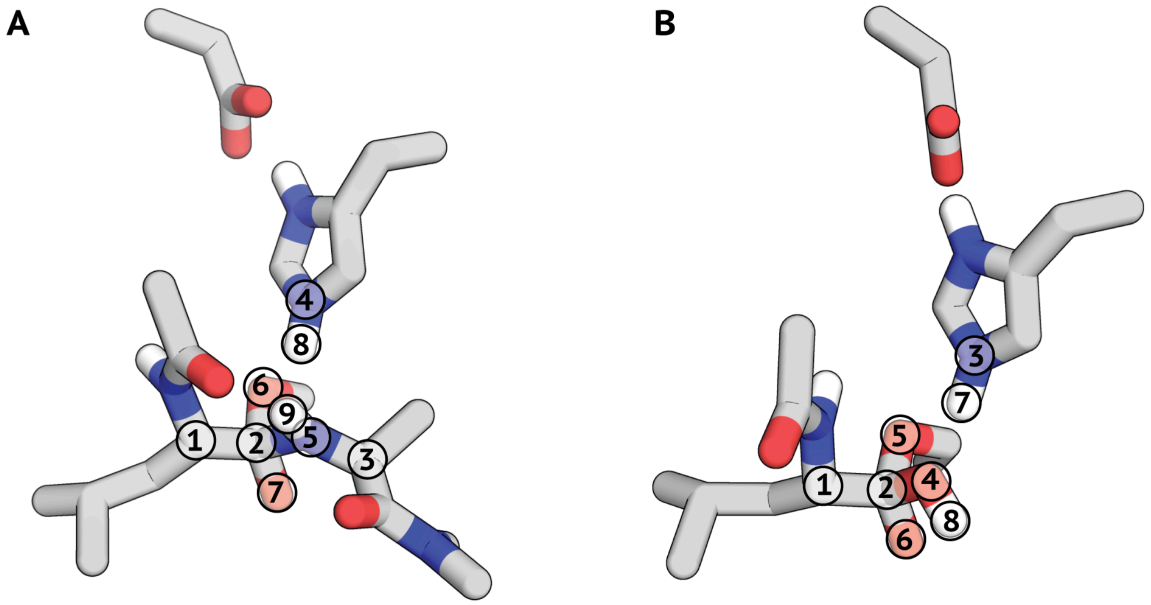

Figure S9. **Atoms used in defining the input layer of DeepTDA variables.** All pairwise distances constitute the input layer. Numbers are used in Figures S10,11 and in Plumed input files. **A.** Acylation stage definition. **B.** Deacylation stage definition. Shown is the intermediate (INT) state.

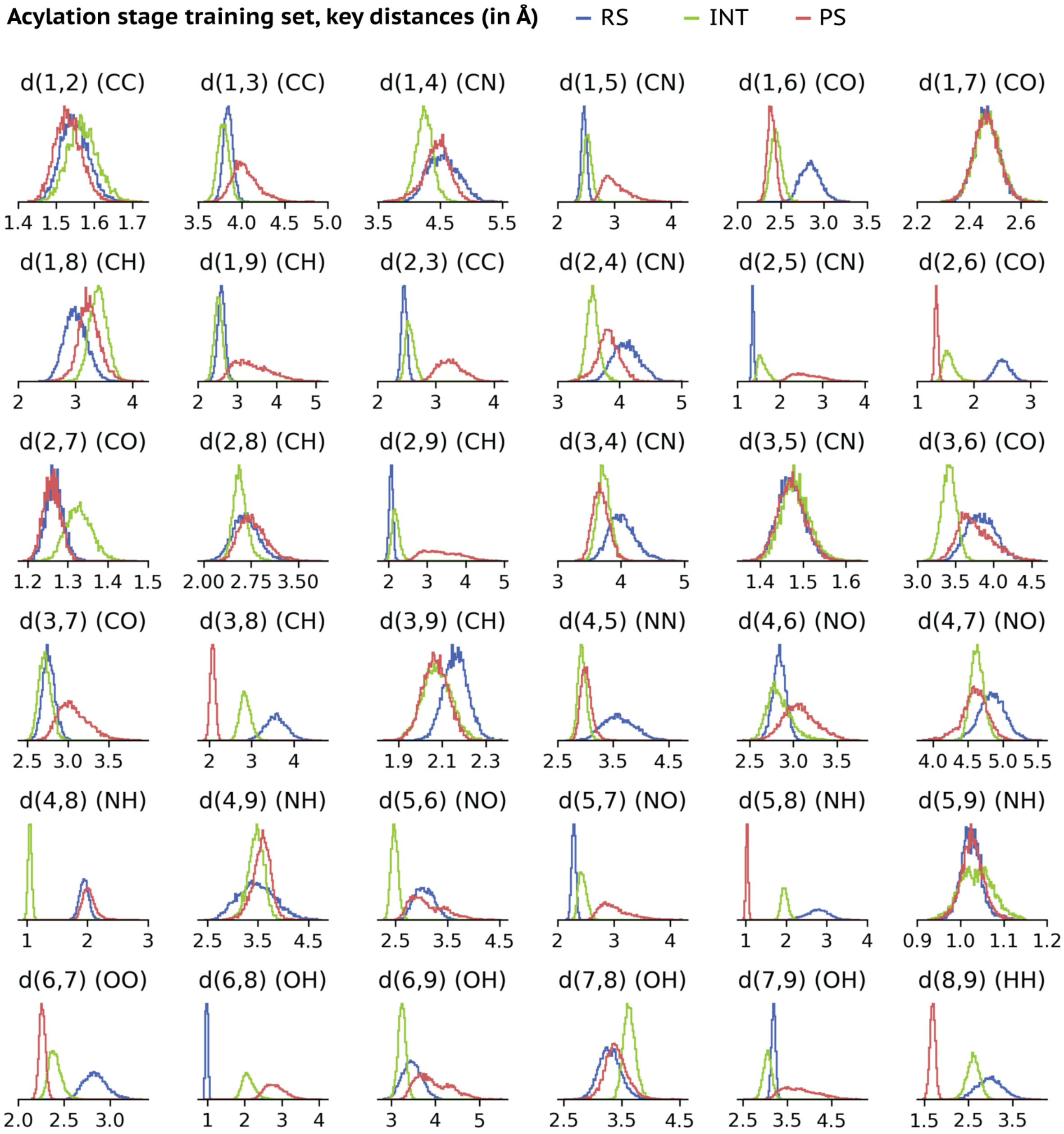

Figure S10. **Distributions of all input layer variables used to construct the acylation DeepTDA CV.**

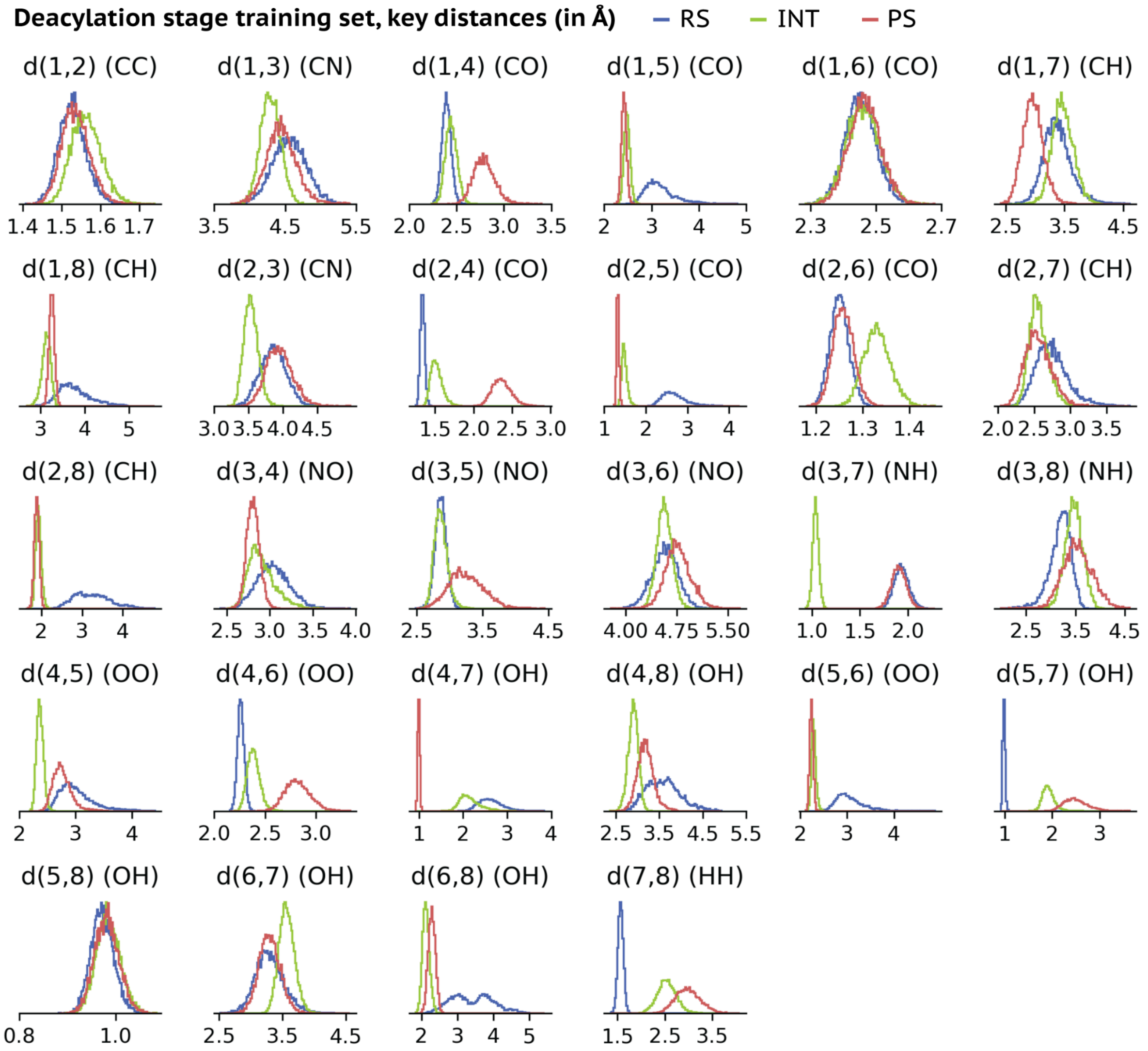

Figure S11. **Distributions of all input layer variables used to construct the deacylation DeepTDA CV.**
